# Design of modular gellan gum hydrogel functionalized with avidin and biotinylated adhesive ligands for cell culture applications

**DOI:** 10.1371/journal.pone.0221931

**Published:** 2019-08-30

**Authors:** Christine Gering, Janne T. Koivisto, Jenny Parraga, Jenni Leppiniemi, Kaisa Vuornos, Vesa P. Hytönen, Susanna Miettinen, Minna Kellomäki

**Affiliations:** 1 Faculty of Medicine and Health Technology, BioMediTech, Tampere University, Tampere, Finland; 2 Research, Development and Innovation Center, Tampere University Hospital, Tampere, Finland; 3 Fimlab Laboratories, Tampere, Finland; University of Vermont, UNITED STATES

## Abstract

This article proposes the coupling of the recombinant protein avidin to the polysaccharide gellan gum to create a modular hydrogel substrate for 3D cell culture and tissue engineering. Avidin is capable of binding biotin, and thus biotinylated compounds can be tethered to the polymer network to improve cell response. The avidin is successfully conjugated to gellan gum and remains functional as shown with fluorescence titration and electrophoresis (SDS-PAGE). Self-standing hydrogels were formed using bioamines and calcium chloride, yielding long-term stability and adequate stiffness for 3D cell culture, as confirmed with compression testing. Human fibroblasts were successfully cultured within the hydrogel treated with biotinylated RGD or biotinylated fibronectin. Moreover, human bone marrow stromal cells were cultured with hydrogel treated with biotinylated RGD over 3 weeks. We demonstrate a modular and inexpensive hydrogel scaffold for cell encapsulation that can be equipped with any desired biotinylated cell ligand to accommodate a wide range of cell types.

## Introduction

Biomaterials are essential instruments in the field of tissue engineering and regenerative medicine that are required to support cells and mimic natural tissue.[1,2] Hydrogels are a class of biomaterials that can simulate the native, physiological, and three-dimensional (3D) environment of mammalian cells and act as an artificial extracellular matrix.[3–8] It has been well-established that 3D tissue matrices must be considered over planar, two-dimensional (2D) surfaces for cell culture applications and *in vitro* disease modeling.[5] The biomaterial should recreate all aspects of the natural cell environment, including dimensionality, physical, mechanical, and biochemical properties. These properties are then engineered to control cell attachment and cell fate.

There are several issues that need to be considered when designing a hydrogel for cell culture applications. Foremost, all components must assert their biocompatibility and the final material, as well as the reagents used in the preparation method, must be non-toxic and elicit no negative cell response.[1,5] However, rather than only providing a passive environment, hydrogels are also required to promote certain cell functions and support cell recognition and response.[5] In practice, the hydrogel should contain cell recognition moieties, such as peptide sequences or proteins like growth factors, i.e., sites that actively guide cell response. In a similar fashion, the mechanical properties and degradation profile of the hydrogel matrix must actively influence the response of the seeded cells.

Further, the stiffness of the microenvironment is directly conveyed to the cytoskeleton through cell attachment and integrin signaling. This phenomenon, called mechanotransduction, is known to affect the differentiation of cells[8–10], cell attachment, and migration.[5,11,12] In turn, mechanotransduction requires the integrin ligand to be strongly tethered to the polymeric network. This attachment is necessary to promote cell spreading and to prevent the diffusion of the cellular cues.[4]

Finally, there are a few more technical issues of hydrogel design that include the manipulation and handling as well as the availability and cost-efficiency of the material.[13] Clearly, the material must be affordable to enable its use on a more general basis. The ability to handle, transport, and analyze the final hydrogel product has definite practical advantages over more sensitive constructs. Ultimately, the design of a hydrogel must strike a balance between functional complexity and technical simplicity.

One approach to the design of hydrogels for tissue engineering applications is the conjugation of bioactive molecules to the polymer. Examples of conjugation techniques include the formation of zero-length bonds, bio-orthogonal coupling, and the use of protein-ligand binding. Zero-length bonds form a short, direct chemical bond between the polymer and the coupled compound that are achieved using carbodiimide coupling and thiol-based conjugation techniques.[14] The so-called bio-orthogonal coupling, such as the strain-promoted azide-alkyne cycloaddition[15], do not interfere with compounds found in living organisms. Here, we exploit the protein-ligand binding, which provides a simple and native tool to form substrate-ligand complexes. Certain proteins have the ability to bind small, specific molecules with high affinity and selectivity. One of the most in-depth studied protein systems is the avidin-biotin binding system.[16–18] Avidin is a protein with the capability to bind biotin with outstanding selectivity and specificity. The avidin-biotin interaction is deemed to be the strongest non-covalent bond in nature with a dissociation constant of K_d_ ~10^−15^ M, and it has often been used in biochemical assays, diagnostics, and tissue engineering.[18] Furthermore, this type of protein-affinity system has been exploited for many different applications in chemistry, biosciences, and tissue engineering. Indeed, modular approaches, such as the use of nanocellulose for 3D printing [19], 2-hydroxyethyl methacrylate flat substrates[20], agarose for spatial patterning [21], and porous poly-L-lactic acid scaffolds [22], have been presented and will be discussed in chapter 4.1.

The base polymer used in this study is gellan gum (GG), which is an anionic polysaccharide that has previously been investigated for various cell culture applications.[23–26] GG is able to form self-supporting hydrogels, that do not flow and fracture under high stress and also described as “true gels” in contrast to “weak gels”.[27] To form true hydrogels, GG is hereafter ionically crosslinked with the bioamine spermidine. In its native state, GG has been found to be biocompatible, but most cell types do not readily adhere to or favor the material. Previously, our research group established that functionalization of gellan gum with ECM proteins is needed to obtain better cell attachment and migration.[24]

Here, we propose the coupling of the avidin protein to gellan gum to create a modular material that can be modified through the addition of biotinylated cell cues. The cues can enable cell attachment, guide differentiation, or even present drug molecules. In this study, we use an avidin analogue called charge-neutralized chimeric avidin, which has been developed and produced by our group. It has identical affinity for biotin, but increased stability against pH and temperature treatment compared to wild-type avidin.[28] Furthermore, we have sought suitable applications for the charge-neutralized chimeric avidin in the field of tissue engineering.[29] Biotin is a small organic compound that can be chemically coupled to a desired ligand. Before the gelation step, the hydrogel precursor can be modified without any additional chemical functionalization steps. The choice of biotinylated species can range from attachment factors, such as RGD, to drug molecules, and to growth factors (GF), such as vascular endothelial GF, which also affects differentiation. Ultimately, the goal is to design a non-specialized platform that is adaptable to different applications, while still possessing the innate ability to support cell growth and to allow for the convenient analysis of the tissue engineering construct.

To underline the suitability of the proposed modular hydrogel, we characterized the mechanical properties and avidin functionality of the gellan gum-avidin material. First, GG was purified to remove excess counter-ions, yielding sodium-purified GG (NaGG).[23,30] Then, charge-neutralized chimeric avidin (avd) was coupled to NaGG via carbodiimide conjugation using EDC and NHS[14,23], yielding NaGG-avd. True hydrogels can be formed with suitable crosslinker concentration and compression testing revealed that mechanical behavior was not impaired by the functionalization. Avidin coupled to NaGG is functional and its ability to bind biotin is not impaired by the coupling. Additionally, avidin is shown to be covalently coupled to the polymer network as it does not diffuse from the gel. Human fibroblasts were cultured in NaGG-avd and their viability was assessed. Similarly, the effect of NaGG-avd on human bone marrow-derived stem cells was studied for a total culture time of 3 weeks, highlighting the long-term stability of the gels.

## Materials and methods

All materials were acquired from Sigma-Aldrich, if not otherwise stated. Charge-neutralized chimeric avidin (avd) was kindly donated by the Protein Dynamics group at the Tampere University and is commercially available for research use at Ref. [29].

### Purification

Gellan gum (Gelzan^TM^, low acyl, M_w_ 1 kg/mol) was purified to remove counterions in the product and to replace them with sodium ions.[30,31] Briefly, a gellan gum solution (0.5% w/v, 400 mL) in dI water (milliQ) was combined with Dowex cation exchange resin (5 g, H+ form, 50–100 mesh, prerinsed in HCl) and stirred for 30 min at 60 °C. The exchange resin was removed from the solution through decantation and the pH was adjusted to 7.5 with NaOH. The purified product was then precipitated in *i*-propanol and lyophilized over 2 days.

The ion concentration of NaGG was determined with inductively coupled plasma optical emission spectrometry (ICP-OES). Hence, a part of the sample was digested in sulfuric acid and hydrogen peroxide following the protocol by Kirchmajer et al. (2014)[31] and measured with Agilent 5110 ICP-OES (Agilent Technologies).

### Functionalization and structural characterization

The functionalization reaction was based on the publication by Ferris et al. who similarly activated gellan gum with 1-ethyl-3-(3-dimethylaminopropyl)-carbodiimide (EDC) and N-hydroxysuccinimide (NHS) to couple the peptide sequence RGD.[23] A solution of sodium-purified gellan gum (NaGG, 10 mg/mL, 10 or 20 mL) was dissolved in HEPES buffer (50 mM, pH 6.5) and stirred at 40 °C. The gellan gum was then activated with EDC (0.4 M) and sulfo-NHS (1.0 M) for 15 min and consequently quenched with *β*-mercapthoethanol (28 μL, final concentration 20 mM). Finally, charge-neutralized chimeric avidin (1 mg/mL, 3.5 mL in HEPES 50 mM, pH 6.5) was added and the mixture was stirred for 5 h at 40 °C. The functionalized product was dialyzed over 5 days (MWCO 12–14 kDa) and subsequently lyophilized. Two different batches of NaGG-avd were studied. Each batch differed in the amount of avidin used and the final avidin concentration in the material. Here, these modular hydrogels are termed NaGG-avd(L) for low avidin concentration (4 mg avidin/ 1 g NaGG) and NaGG-avd(H) for high avidin concentration (21 mg avidin/ 1 g NaGG).

**Fig 1:**
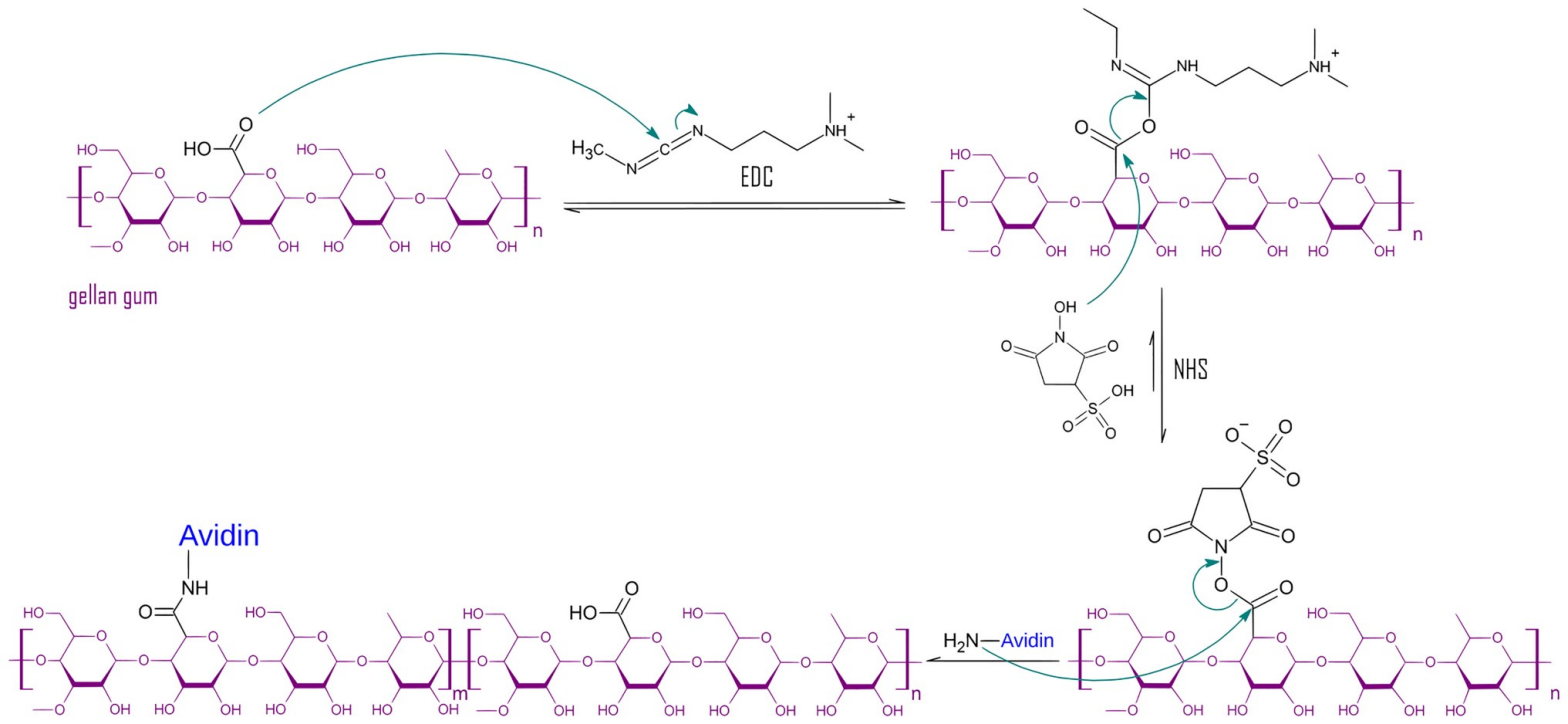
Reaction scheme of NaGG functionalization with avidin via activation with EDC and NHS. EDC activates the carboxyl group of NaGG, and the intermediate is stabilized with NHS to prevent activation of avidin with EDC. The final product is a combination of functionalized [m] and non-functionalized [n] NaGG repeating units.

The reaction scheme of the functionalization is shown in Fig 1. The unstable intermediate of the active ester is stabilized through the addition of NHS, which forms an amine-reactive sulfo-NHS ester. The primary amines of avidin react with the stable intermediary to form a peptide bond. Notable side reactions include the activation of the carboxyl bonds present in the protein structure of avidin. It can, however, be assumed that EDC quickly deactivates through hydrolysis in aqueous media.

The resulting structure was investigated by means of avidin-biotin binding and fluorescence spectroscopy as well as with electrophoresis (SDS-PAGE with urea) and elution analysis. A fluorescence titration curve was prepared from NaGG-avd (1 mg/mL in HEPES pH 7, 10% sucrose) and the biotinylated fluorescent dye b5F (biotin-5-fluorescein, 2 μM in DMSO and PBS). Control curves were prepared with NaGG and NaGG-avd blocked with biotin (3 μL, 0.17 mg/mL in 50 mM sodium-phosphate, 100 mM NaCl buffer, pH 7). Aliquots of 25 μL b5F were added to a 2 mL sample in a cuvette and each measured after 2 min with a QuantaMaster PTI spectrofluorometer (Photon Technology International, Inc., Lawrenceville, NJ, USA) (excitation at 495 nm, emission at 520 nm, slits 2 nm).

For elution analysis, hydrogel samples were prepared with 5 mg/mL NaGG-avd and 10 μM b5F (14 μL/mL NaGG-avd) and crosslinked with 0.5 mg/mL spermidine (SPD). The samples were incubated in the mold overnight and then placed into 500 μL PBS for up to 48 h in an incubator under shaking at 37 °C. The eluate was taken at time points of 1 h, 6 h, 24 h, and 48 h, and the fluorescence intensity was measured. As a reference sample, NaGG was mixed with avidin without conjugation and gels were formed as described above.

Sodium dodecyl sulfate polyacrylamide gel electrophoresis (SDS-PAGE) was used to further examine the covalent nature of the NaGG-avd conjugate through comparison with a non-covalent mixture of NaGG and avidin (avd). To improve the resolution of the SDS-PAGE, urea was added to the samples and the cast gel. The PAGE gel was cast according to standard procedure, but urea (8 M) was added. All samples were incubated with biotin (85 μg/mL final concentration) to stabilize the avidin tetramer. After addition of loading buffer, the samples were heated to 50 °C for 15 min. After a short cooling period, 8 M urea was added to each sample. The electrophoresis was performed at +4 °C for 3 h at 100 V. Finally, the gel was stained with Oriole^TM^ fluorescent gel stain (Bio-Rad) and imaged with a ChemiDoc MP imaging system (Bio-Rad Laboratories) with Image Lab software.

### Physical properties

Hydrogel samples were prepared with a uniform mixing technique, which has been described earlier^30^, to yield homogenous, disc-shaped samples crosslinked with the cationic compound SPD. Gellan gum (5 mg/mL in HEPES/sucrose solution, pH 6.5) was mixed with spermidine (0.5 mg/mL) in 5:1 volume ratio. If CaCl_2_ was used instead of spermidine crosslinker, a concentration of 10 mM was added in same volume ratio. The hydrogel solution was then warmed in a mixing vial under constant stirring (300 rpm) and the crosslinking solution was added. The solution was then swiftly transferred to the mold before the true gel was formed.

Compression behavior was analyzed with a Bose BioDynamic ElectroForce Instrument 5100 using WinTest 4.1 software (TA Instruments, USA). Disc-shaped samples with a diameter of 1.2 cm and a height of 4.5 mm were prepared in PP/PE molds, and the number of parallel samples (n) was 5. The test was carried out as uniaxial, unconfined compression in air at ambient pressure and temperature. The sample was prevented from sliding with wet cellulose paper and compressed with a speed of 10 mm/min to 65% of the original sample height. From the resulting stress-strain curve, the fracture strain and fracture strength were analyzed.

The swelling behavior of the functionalized hydrogel NaGG-avd was monitored over 3 weeks. The chosen hydrogel compositions for the swelling corresponded to the studied to compression samples. The gels were incubated in 500 μL of PBS or DMEM F-12 at 37°C for up to 3 week. The samples were then weighed for their wet weight and consecutively lyophilized and weighed again to determine the dry mass. (Data shown in S4 Appendix).

### Cell culture

Human fibroblasts WI-38 (passage 24–26, Sigma-Aldrich/Culture Collections, Public Health England) we expanded for one week in WI-38 medium, consisting of 10 vol% FBS, 25 U/mL penicillin/streptomycin (pen/strep; Lonza, Basel, Switzerland) in DMEM-F12 1:1 (Gibco, Thermo Fisher Scientific, Waltham, MA) until confluent. The hydrogel solutions of NaGG and NaGG-avd were sterilized prior to cell culture through filtration (Whatman^®^ FP30/0.2 CA-S). The cells were seeded on top of the hydrogel (2D, 63 000 cells/cm^2^) and encapsulated in the hydrogel (3D, 950 000 cells/mL) using a Greiner Cellstar 48-well plate (Sigma-Aldrich). The samples were prepared similarly as described for the compression test sample, where the NaGG-avd solution was heated and stirred in a mixing vial at 37 °C.[32] For cell culture purposes, the biotinylated compound was added, followed by the cell suspension and mixed gently (300 rpm) to achieve homogeneous 3D distribution. Finally, the crosslinker spermidine (0.5 mg/mL, 17 vol% of NaGG-avd) was added and the hydrogel mixture was cast into the well-plate.

The tested material compositions included NaGG-avd with biotin (0.17 mg/mL in 50 mM sodium-phosphate, 100 mM NaCl buffer, pH 7), biotinylated cyclic RGD (cyclo[Arg-Gly-Asp-D-Phe-Lys(Biotin-PEG-PEG)] 0.1 mg/mL in H_2_O, 0.3 μg/mL in final gel), and biotinylated human fibronectin (bFN, 2.52 mg/mL, 33 μg/mL in final gel; fibronectin was purified by gelatin affinity chromatography from outdated plasma preparation and chemically biotinylated). The concentration of biotinylated species was set to match the number of avidin binding sites determined with fluorescence titration. As a reference, the fibroblasts were grown on the well-plate (PS) bottom. The cells were then cultured for 3 days and LIVE/DEAD^®^ stained.

The cell culture samples were stained using the LIVE/DEAD^®^ viability/cytotoxicity assay (Molecular probes, Thermo Fisher Scientific) containing calcein acetoxymethyl ester (Ca-AM) and ethidium homodimer-1 (EthD1). The dyes were diluted in PBS (Lonza) (final solution concentration Ca-AM 0.8 μM and EthD1 1.0 μM) and added on top of the cell culture samples. The samples were incubated for 30 min at room temperature and imaged with an Olympus IX51 inverted microscope and an Olympus DP30BW digital camera (Olympus, Tokyo, Japan). The images were then analyzed with ImageJ software (U.S. National Institutes of Health, Bethesda, MD)[33] through the particle counting algorithm. The cell viability was determined from the area according to Eq (1), while cell spreading was determined from the same data using Eq (2):

$$\text{Viability}=\frac{\text{area}\;\text{of}\;\text{live}\;\text{cells}}{\text{area}\;\text{of}\;\text{live}\;\text{cells}+\text{area}\;\text{of}\;\text{deadcells}}$$ (1)

$$\text{Spreading}=\frac{\text{area}\;\text{of}\;\text{live}\;\text{cells}}{\text{image}\;\text{area}}$$ (2)

Primary human bone marrow stromal cells (hBMSC) were previously harvested, isolated, and cryo-preserved in gas phase nitrogen by the Adult Stem Cell Group, BioMediTech, Tampere University, in accordance with the Regional Ethics Committee of the Expert Responsibility area of Tampere University Hospital, ethical approval R15174. The hBMSCs were isolated from an anonymous donor (labeled 6/16) with the patient’s written informed consent during surgery at the Department of Orthopaedics and Traumatology at Tampere University Hospital. The isolation of hBMSCs was performed as described previously with slight modifications.[34,35] Briefly, the bone marrow aspirate was rinsed with DPBS (Lonza), resuspended in Ficoll (GE Healthcare, Chicago, IL, USA), centrifuged 800 g for 20 min at room temperature after which mononuclear cells were collected, washed twice with α-MEM (Gibco, Thermo Fisher Scientific) and centrifuged 400 g for 15 min at room temperature. In the following, cells were seeded into PS flasks (Nunclon; Sigma-Aldrich) in basic medium containing 5% HS (Biowest, Nuaillé, France) in α-MEM and 1% 100 U/mL pen/strep and 5 ng/mL hFGF-2 (Miltenyi Biotec, Bergisch Gladbach, Germany), and expanded until 80% confluence. The isolated hBMSCs were characterized with flow cytometry (S6 Appendix).

The hBMSCs (passage 6) were thawed and expanded for one week in T75 PS flasks (Nunclon; Sigma-Aldrich) in basic medium until confluent. The cells were then harvested and seeded into the hydrogel via uniform mixing (cell density 950 000 cells/mL), as described for the fibroblast test using spermidine as crosslinker. The studied materials were NaGG (as a reference) and NaGG-avd with the addition of biotinylated cyclic RGD (2.5 μM final solution concentration). The cells were cultured for 21 days in the hydrogels with the addition of osteogenic medium (5 vol% HS, 0.25 mM ascorbate-2-phosphate, 10 mM *β*-glycerophosphate, 1% 100 U/mL pen/strep in α-MEM, with the addition of dexamethasone 5 nM), which was replaced every other day.

Viability assay was carried out on day 3, 14, and 21 similar to the WI-38 experiment. Ca-AM and EtHD-1 were diluted in PBS (final solution concentration Ca-AM 0.5 μM and EthD1 0.25 μM) and added on top of the cell culture samples. On day 21, the samples were stained with phalloidin (0.17 μg/mL in 1% bovine serum albumin BSA) and 4’,6-diamidino-2-phenylindole (DAPI; dilution 1:2000 in PBS; Sigma-Aldrich). The samples were then fixed (0.1% Triton x-100 and paraformaldehyde PFA) and blocked (1% BSA) before staining.

## Results

GG was purified to remove counterions (Ca^2+^, K^+^, Mg^2+^) and to replace them with sodium (Na^+^). ICP-OES data can be found in S1 Appendix. The result of the ion analysis shows that the calcium content was reduced to below 0.1 wt%, closely matching reported literature values ^24,29^. NaGG was successfully functionalized with the recombinant protein ‘charge-neutralized chimeric avidin’ (avd) through carbodiimide conjugation. NaGG-avd solution was prepared in HEPES buffer with sucrose. Hydrogel samples were prepared through gelation with SPD or CaCl_2_ overnight in disc-shaped molds with dimensions of about 4.1 mm in height and 11.6 mm in diameter.

### Success of functionalization

**Fig 2:**
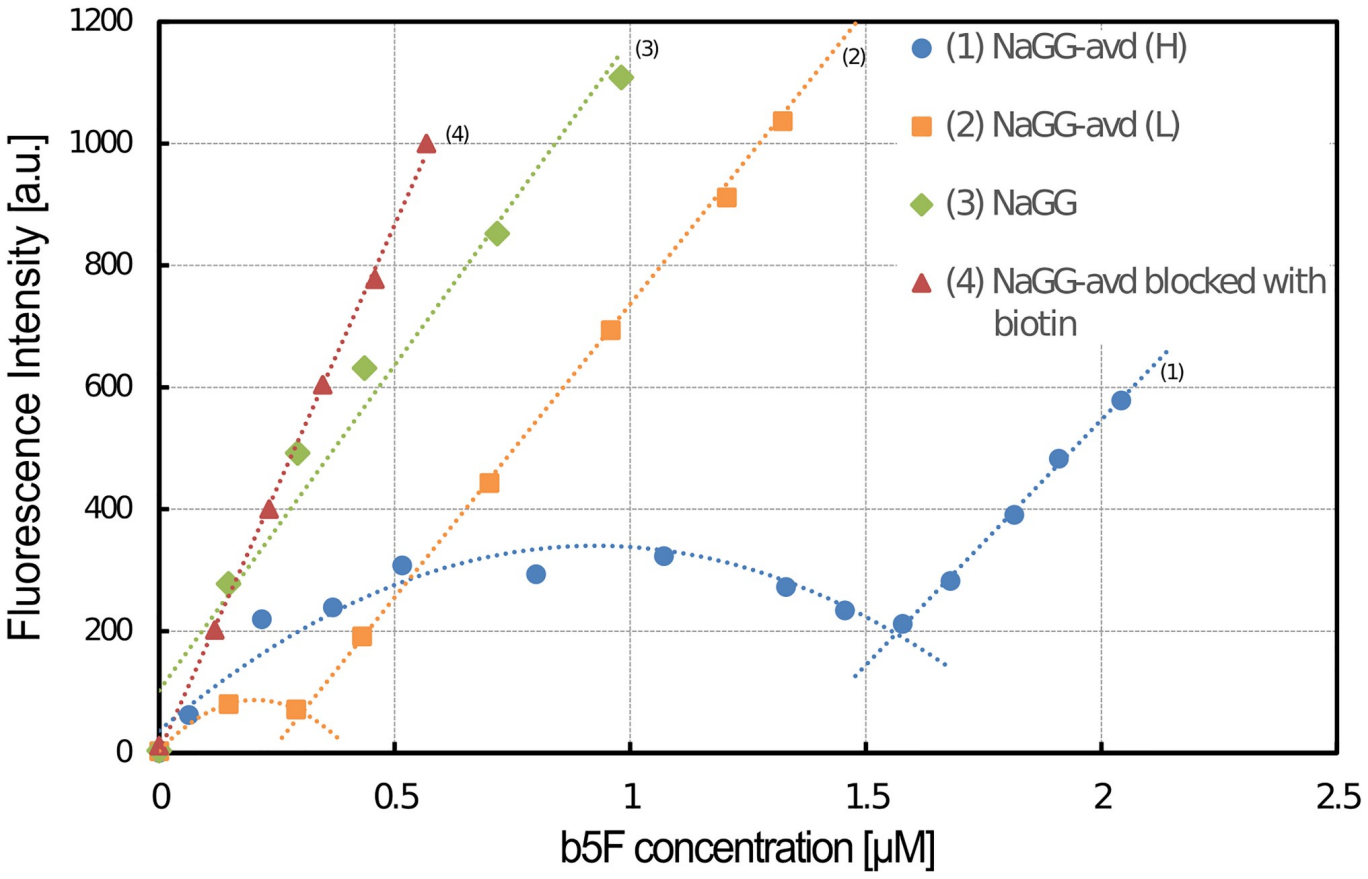
Fluorescence titration curves with step-wise addition of b5F. Non-functionalized NaGG (green) and functionalized NaGG-avd blocked with biotin (red) show a linear curve, whereas functionalized NaGG-avd (yellow and blue) with available biotin-binding sites show quenching behavior at low b5F concentration. From the intersection of quenched and linear parts the biotin binding site concentration can be deduced. From the intersection or linear and polynomial curves, the biotin-binding concentration was calculated.

Fluorescence analysis was carried out in different ways to prove the binding and functionality of avidin. The biotinylated fluorescence dye biotin-5-fluorescein (b5F) was used in all cases. Fluorescence titration with b5F was carried out in order to confirm that avidin retains its ability to bind biotin after being coupled to gellan gum. Therefore, small amounts of b5F were added to the analyte. This dye shows a quenching effect to approximately 50% of fluorescence strength when bound to an avidin specific binding site, which results in a non-linear curve. The concentration of available biotin binding sites can be derived from the intersection of the quenched curve and the linear region after saturation. Fig 2 shows the curves of coupled NaGG-avd, unmodified NaGG, and coupled NaGG-avd that has been blocked with biotin. The unmodified and blocked samples show a linear increase in fluorescence intensity, whereas the avidin-modified samples show a quenched curve followed by a linear increase.

From the titration curves in Fig 2, the avidin concentration was estimated to be 0.075 (L) and 0.375 (H) μM in 1mg/mL NaGG-avd. The degree of functionalization (d_funct_) can be calculated as the molar ratio between avidin-functionalized GG repeating units [n] and non-functionalized repeating units retaining the carboxyl (COOH) group [m]. The indices n and m refer to the reaction scheme in Fig 1, and the functionalization degrees are derived from Eq (3):

$$d_{funct}=\frac{\left[n\right]}{\left[m\right]}=\frac{mol(avidin)}{mol(GG-COOH)}∙100\text{\%}$$ (3)

A functionalization degree of 0.005 mol% (L) and 0.027 mol% (H) was achieved between different functionalization degrees, assuming one avidin is bound to only a single carboxyl group.

**Fig 3:**
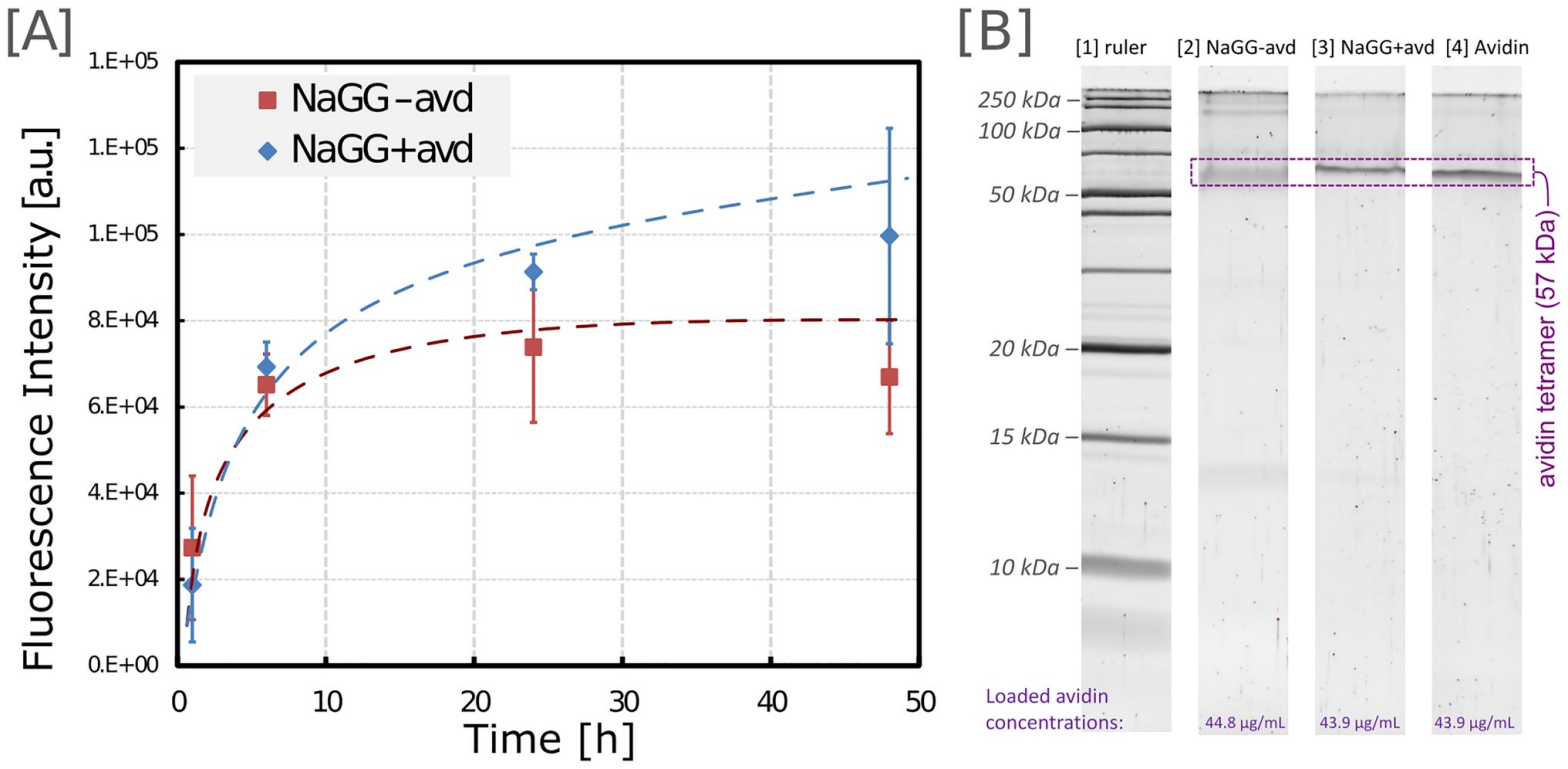
Avidin-GG coupling analysis. (A) Elution analysis: Fluorescence intensity of supernatants of hydrogel samples immersed in PBS. Supernatant was collected from separate samples over 2 days and analyzed with fluorescence spectrometer. Error bars represent standard deviation calculated from three independent samples, and logarithmic fit curves were added to guide the eye. (B) SDS-PAGE: Electrophoretic separation and comparison of conjugated (NaGG-avd) and unconjugated (NaGG+avd) samples. Tetrameric avidin band (57 kDa) is very faint and blurry for NaGG-avd, compared with NaGG+avd and pure avd, indicating covalent bonding between avidin and polymer.

To ascertain the covalent binding between gellan gum and avidin, hydrogel samples were prepared containing b5F. The samples were incubated in phosphate buffered saline (PBS) at 37 °C, with separate samples for each time point of 1 h, 6 h, 24 h, and 48 h, and the fluorescence intensity was measured. As a reference sample, NaGG hydrogel samples were prepared with the addition of a comparable amount of avidin. Fig 3(A) shows the resulting graph.

Initially, the fluorescence intensity of both eluates is very similar, alluding to a leaching of whole polymer chains from the gel sample carrying the b5F. However, after roughly 24 h in suspension, the fluorescence intensity of NaGG-avd starts to reach a plateau and virtually no further b5F leaves the gel. In contrast, the reference sample NaGG+avd continues to exude b5F, and after 48 h the fluorescence intensity of the eluate is considerably higher.

When choosing methods to investigate the gellan gum-avidin bonding, it was surmised that ^1^H-NMR does not sufficiently distinguish the newly formed carbodiimide-coupled peptide bond from the already present bonds in the studied biomacromolecule. Similarly, FT-IR spectroscopy is unable to show the bond characteristics between the two macromolecules. Thus SDS-PAGE was the method of choice to confirm the gellan gum-avidin binding. The urea PAGE shows a strong band for avidin tetramer in the reference sample (NaGG+avd) and pure avidin, but a faint, diffuse band for the conjugated sample (NaGG-avd) in Fig 3(B). The dispersion of the band may be due to avidin coupled with short GG chain fragments, leading to a wider molar mass range. A very faint band for the avidin monomer can be observed for NaGG-avd, but not for the reference sample NaGG+avd. GG itself has a molecular weight of around 1000 kDa and does not appear as a band on the SDS-PAGE. The full uncropped and un-altered blot and gel image is available in S2 Appendix. A simple washing test was carried out is described in S8 Appendix, demonstrating a 4-fold increase in retained biotinylated fibronectin when comparing unfunctionalized (NaGG) and functionalized GG (NaGG-avd). This further underlines the functionality of the proposed modular hydrogel.

### Physical properties

**Fig 4:**
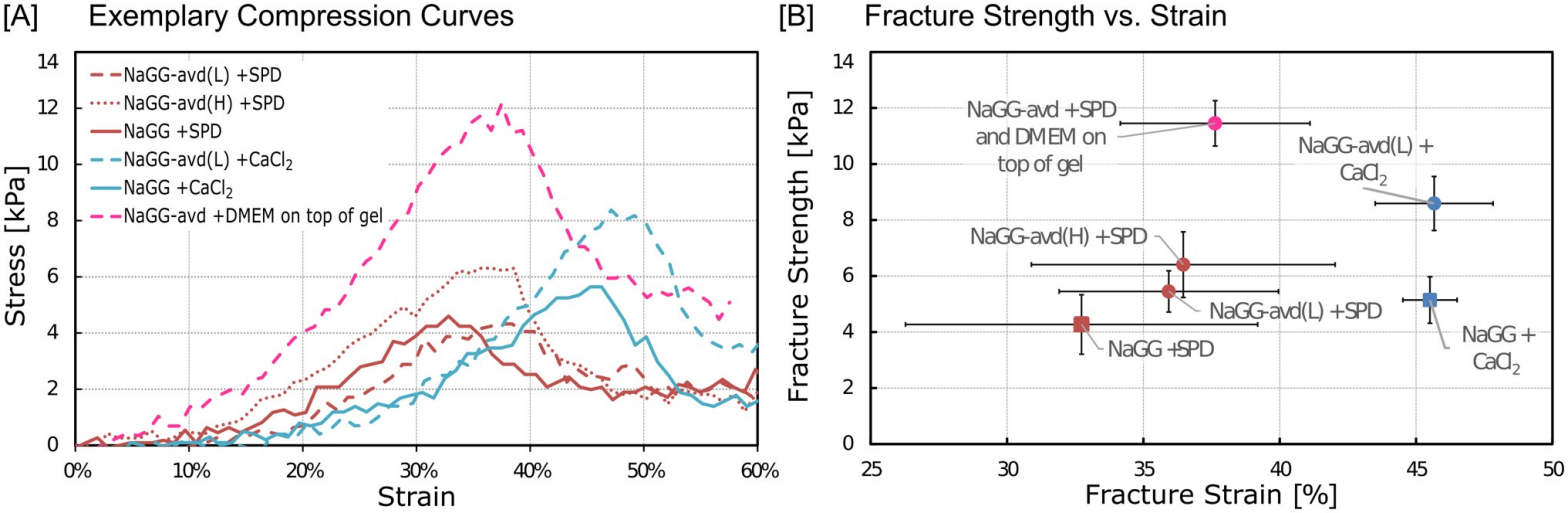
Compression testing results. (A) Representative compression curves of samples with different composition. (B) plot of fracture strength against fracture strain When examining all data, fracture strength (p = 9.28 x10^-11^) and fracture strain (p = 1.4 x10^-4^) are statistically different. Testing within samples formed with SPD, however, no significant difference is observed between fracture strength (p = 0.06) and fracture strain (p = 0.54). Error bars represent one standard deviation calculated from five independent samples.

The gelation time can be estimated through a qualitative tube-tilt test. NaGG-avd forms true gels during a period of about 30 seconds to one minute after the hydrogel has been transferred to the mold. For mechanical testing, the hydrogel samples were prepared as described earlier and incubated overnight at 37 °C before testing. The samples were compressed once to 65% of their original height and then discarded. Representative compression curves for each sample type are shown in Fig 4(A). The results of fracture strength and strain are plotted for comparison in Fig 4(B) and shown in S3 Appendix.

From Fig 4, a significant difference in fracture point between SPD and CaCl_2_ gels can be seen. Hydrogels formed with CaCl_2_ are more elastic and have a higher fracture strain of ~46%, while gels formed with SPD are more brittle and have fracture occurring already at around 35% strain. However, the fracture strength values for either crosslinking method are similar. The extensive error exhibited by the strain value may be due to a manual error when positioning the sample on the compression piston. One-way analysis of variance (ANOVA) was performed assuming a confidence level of 95% (p < 0.05) with Microsoft Office Excel. The analysis showed that there is a significant difference between all shown samples (p < 0.05), but the samples prepared with SPD showed no significant difference (p = 0.07).

Remarkably, it appears that the addition of avidin to the polymer consistently increases both the fracture strength and fracture strain of the hydrogel samples. The samples were prepared in a buffer at pH 6.5, whereas during cell culture experiments the pH was at 7.4 determined by the cell culture medium. Although charge neutralized chimeric avidin has an isoelectric point (pI) of 6.92 (theoretical value), it can be assumed that the terminal N-acetyl glucosamine, as well as lysine and arginine groups, will carry a positive charge within the range of the mentioned pH values regardless. These positively charged amino groups aid in the crosslinking of the anionic gellan gum as additional crosslinker via charge screening. This is beneficial as the net effect of avidin functionalization is increased mechanical toughness.

An initial test with cell culture medium (DMEM) was carried out to observe the behavior of the gels in cell culture conditions. The hydrogel was crosslinked with SPD, and DMEM was added on top after the gels were formed. The samples were then incubated overnight before testing. The cationic species in DMEM diffuse into the hydrogel and increase the fracture strength of the gels significantly.

The swelling test showed no swelling of the gels, but rather a contraction over time. On average, the gels lost 3% of their original mass over 3 weeks. The results of the swelling test can be found in S4 Appendix.

### Cell culture

**Fig 5:**
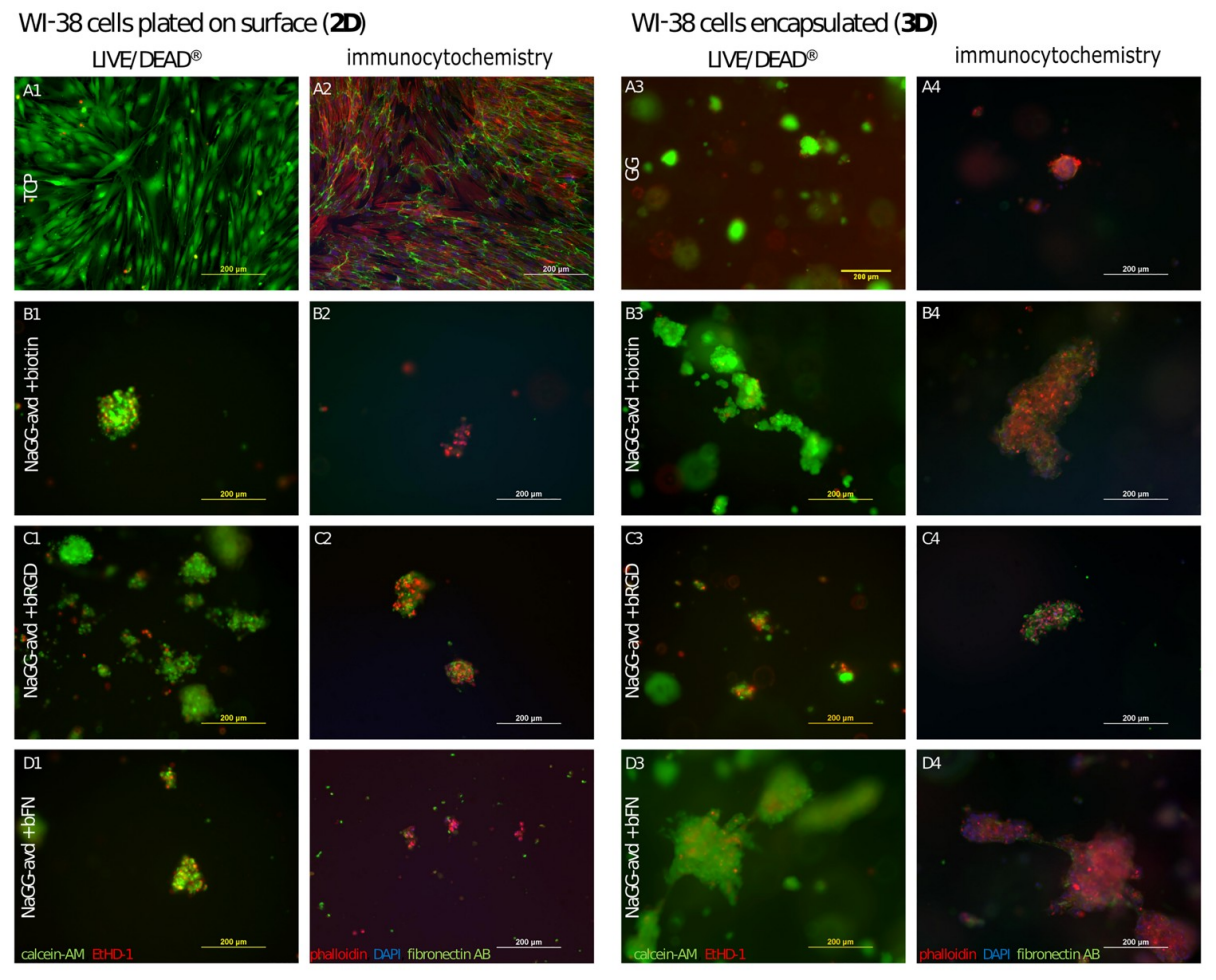
WI-38 fibroblasts after three days in culture. A1-D2 (left side): 2D culture with 63 000 cells/cm^2^, A3-D4 (right side): 3D culture with 950 000 cells/mL in gel. Images A1-D1 and A3-D3 LIVE/DEAD^®^ staining with live cells (Ca-AM, green channel) and dead cells (EthD1, red channel); images A2-D2 and A4-D4 actin filaments (red channel, TRITC-phalloidin), fibronectin (green channel, fibronectin antibody), cell nuclei (blue channel, DAPI).

Human WI-38 fibroblasts were cultured in avidin-modified gellan gum for three days. Three different biotinylated compounds (biotin, RGD, and fibronectin) were added to the hydrogel as separate samples. As control samples, the bare well bottom (TCP, 2D) as well as the unmodified gellan gum (3D) were used. The cells were alive when cultured on top of the hydrogel (2D) as well as when encapsulated in the matrix (3D). Fig 5 shows LIVE/DEAD^®^ fluorescence images after three days in culture (Fig 5A1–5D1 and 5A3–5D3). The images show aggregation to large cell clusters in 2D and 3D. During encapsulation, the 3D distribution of fibroblasts was homogeneous (S9 Appendix). The most notable difference can be observed in the cell distribution and density between unmodified/unpurified GG (control) and the modified hydrogel samples (NaGG-avd). Subsequently, the samples were fixed and immuno-stained (DAPI, phalloidin, fibronectin antibody; Fig 5A2–5D2 and 5A4–5D4). Immunostaining shows no meaningful cell spreading, judging from the shape of the actin cytoskeleton (red). Fibroblasts produced their own fibronectin, as revealed by the fibronectin immunostaining in samples without added bFN.

**Fig 6:**
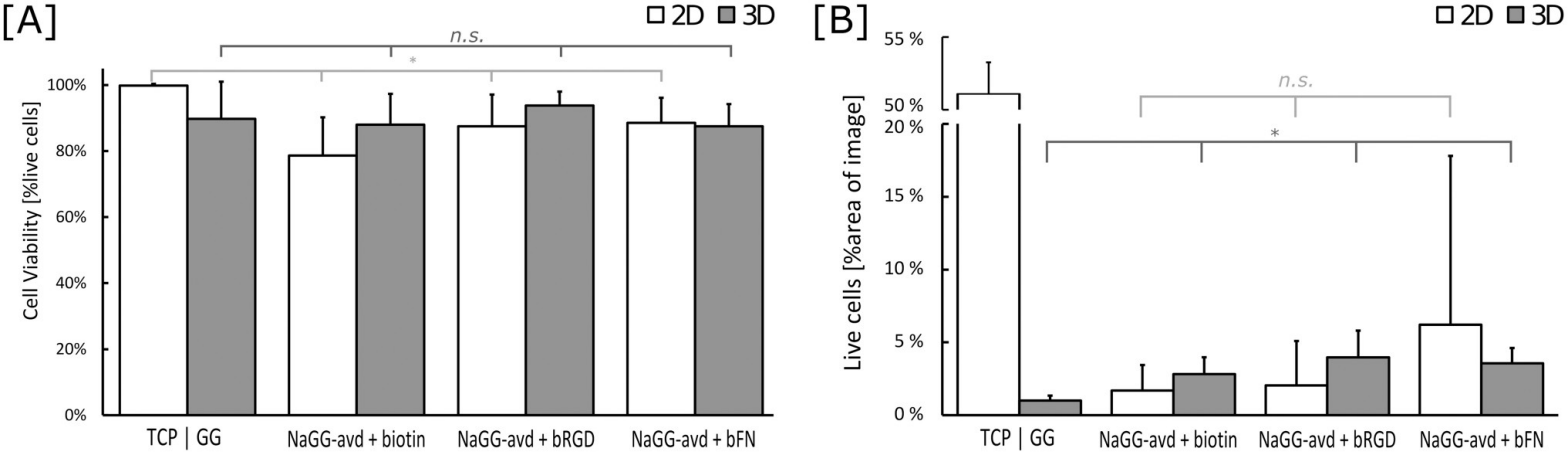
[A] Cell viability and [B] spreading of WI-38 fibroblasts in different materials as calculated from LIVE/DEAD^®^ stain image analysis. Control materials are tissue culture plastic (TCP, 2D) and unmodified, unpurified GG (3D). Bars represent mean values ± SD, n ≥ 10. [A] Cell viability (%live cells vs sum of live and dead cells) demonstrates that all 3D samples (GG, NaGG-avd) are statistically the same (p = 0.09), while the 2D samples (TCP, NaGG-avd) are statistically different (p* < 0.05). Similarly, cell spreading (area% of live cells in image) demonstrates no significant difference between 2D GG based samples (p = 0.20), while there is significant difference between 3D samples (p* = 3.94 x10^-8^).

To analyze the images statistically, images were taken of each well from random areas and the total area of live (green pixels) and dead (red pixels) cells was compared, as shown in Fig 6. Due to strong clustering of cells, the area was used rather than the number of particles. Similarly, the large error, especially for the 2D samples, was caused by aggregation of cells and the large difference of perceived cell density between different images of the same well. As seen in Fig 6A, all 3D samples show viability above 85% but are ultimately very similar with no statistical difference. ANOVA was performed assuming a confidence level of 95% with the result that there is a significant difference between the cell count of all the shown 2D samples, but no significant difference between the 3D samples. Full cell viability data from particle counting analysis is shown in S5 Appendix. Using the area% of the live cell data, cell spreading was analyzed as shown in Fig 6B, referring to the area covered by live cells on the investigated image. ANOVA test reveals no significant difference between cell spreading on or in GG-based samples (GG and NaGG-avd), due to the aforementioned large standard deviation and clustering. There is, however, a significant difference between 3D samples of GG formulations.

**Fig 7:**
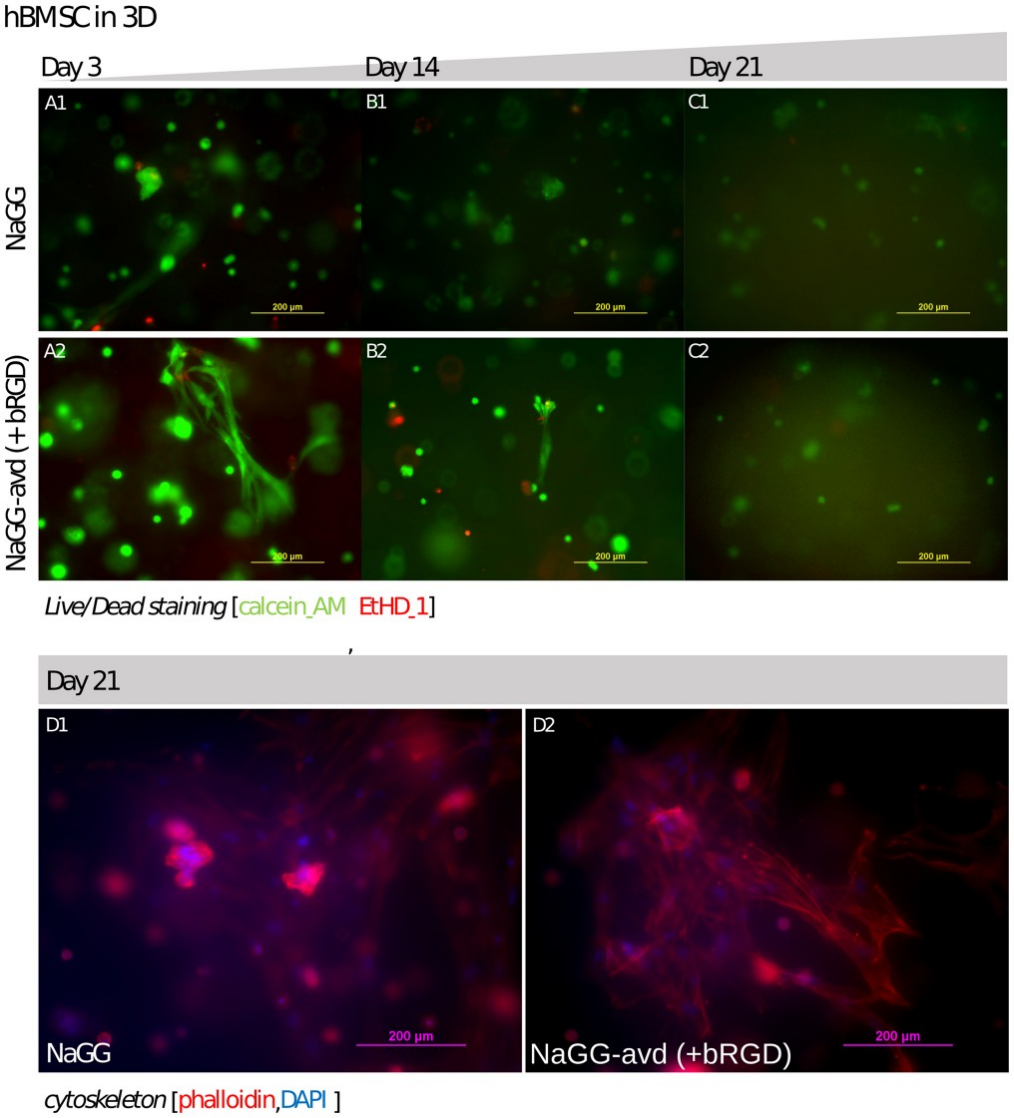
hBMSC encapsulation results (A1-C1 and A2-C2) LIVE/DEAD^®^ stain of hBMSC encapsulated in NaGG (control) and NaGG-avd with bRGD over 21 days. Red stain = dead cells, Green stain = live cells. (D1-D2) Immunocytochemical cytoskeleton staining on day 21. Blue = cell nuclei (DAPI) and Red = actin filaments (phalloidin-TRITC).

Consequently, human bone marrow derived stromal cells (hBMSC) were encapsulated and cultured in NaGG-avd with added bRGD for up to 21 days. Fluorescence images with LIVE/DEAD^®^ stain of the time points 3, 14, and 21 days are shown in Fig 7. After 21 days, the samples were fixed and stained with DAPI (cell nuclei) and phalloidin (actin filaments), referred to as immunocytochemical cytoskeleton staining, as shown in Fig 7D.

The LIVE/DEAD^®^ images demonstrate that the hBMSC are viable in both materials in 3D suspension. Evidently, the image quality degrades the longer the cells are in culture and it becomes difficult to achieve good microscope images due to opaque material. This behavior may indicate a mineralization by the hBMSC differentiating towards bone, but conclusions about differentiation cannot be drawn from actin cytoskeleton alone. In identical culture conditions, stained cell-free blank samples showed no background fluorescence after 21 days (the background image is available in S7 Appendix). Thus, the developing opacity is caused by the cells and is not due to the GG material.

Immunostaining of hBMSC samples at day 21 confirmed the observations of the LIVE/DEAD^®^ images. A majority of the cells appear rounded, but some of them do show elongation. Visibly there are more elongated cells in NaGG-avd+bRGD than in the non-functionalized NaGG, which is more evident with the stained actin (additional cytoskeleton images in S7 Appendix). The hBMSC culture test was analyzed with only staining and microscope images, while no quantitative analysis was carried out.

## Discussion

The presented results support the concept of an avidin-modified hydrogel material for 2D and 3D cell culture applications. We have shown that the functionalized gellan gum can bind biotinylated compounds, thus equipping the hydrogel with bioactive factors and enabling cell attachment. The gellan gum-based material shows little to no degradation and has been found to be feasible for long-term cell culture. Furthermore, it has suitable mechanical properties and convenient preparation.

### Functionalization strategies

There is an abundance of approaches to design hydrogels described in the literature. These approaches include the covalent functionalization of passive polymers with bioactive cues, blending with bioactive native polymers or peptides, and the use of native ECM components.[9,36] A broad selection of materials for 3D cell culture is also available commercially.

Hydrogels prepared from decellularized ECM or ECM components are a popular choice as promoting scaffolds because they naturally contain cell recognition sites. Commercially available examples include PuraMatrix^®^[37], a peptide sequence of arginine, alanine, and aspartic acid, and Matrigel^®^, a reduced growth factor basement membrane matrix.[38] Both materials provide an excellent cell environment and achieve good cell response for a vast range of mammalian cell types. Their mechanical properties are, however, poor. Bulk hydrogels have low stiffness, degrade quickly, and are rather expensive.

On the other hand, there is a plethora of synthetic and natural hydrogel materials that are conjugated with bioactive compounds. These compounds range from small peptide sequences to proteins or other large biological compounds, such as gelatin. An non-exhaustive list of examples includes the conjugation of N-cadherin to alginate[39], conjugation of the peptide sequences RGD and SIKVAV to poly(2-hydroxyethyl methacrylate)[40], or the conjugation of RGD peptide to gellan gum.[23] These attached compounds enhance cell attachment and can guide cell fate. The mechanical properties of the gels are determined by the base polymer and the stiffness can be adjusted to the cell type requirement. However, the synthesis and production of these cell culture materials are elaborate and expensive, while only a narrow application area is targeted. Usually, specific chemistry development is needed in each case of polymer modification. This means that developing a material for neural cell culture does not benefit the needs for hepatic cell culture, while both are still soft tissues.

Even though modular strategies are not as common as single molecule functionalization, avidin-biotin-based approaches have been presented by Kojima (2006), Hobzova et al. (2011), Wylie et al. (2011), and Leppiniemi et al. (2017).[19–22] Kojima et al. (2006) adsorbed avidin onto PLLA disks for the culturing of biotinylated hepatic cells. However, rigid PLLA scaffolds are a very different material type compared with soft hydrogels and their range of application.[22] Leppiniemi et al. (2017) have shown that avidin-functionalized nanocellulose combined with alginate is suitable for 3D printing. In their study, the protein was covalently bound to the cellulose fibrils and confirmed through electrophoresis. Their range of applications included biomedical devices, wearable sensors, and drug-releasing materials for wound healing, and therefore no cytotoxicity assay or cell culture experiments were presented.[19] Nanocellulose forms weak hydrogels without macroscopic hierarchy, and dimensionality was achieved by the addition of alginate and an additional freeze-drying step. On the other hand, gellan gum can form true hydrogels applicable to long-term disease modeling.[41] Further, Wylie et al. (2011) created an advanced system using two different proteins, namely avidin and barnase, for the orthogonal functionalization of agarose hydrogels for 3D patterning. Indeed, the use of a protein-binding phenomenon enables the flexible modification of a 3D cell culture material.[21] Their approach is more complex and encompasses a more time-consuming set-up, whereas our system is applicable to larger scale use and requires no laser equipment. Hobzova et al. (2011) showed the covalent grafting of avidin to planar 2-hydroxyethyl methacrylate (pHEMA) surfaces, and they also compared non-specific surface adsorption and electrostatic interaction as a means of avidin immobilization. Their preliminary cell culture results rudimentarily show the attachment of keratinocytes on surface-modified avidin-pHEMA, but no 3D system or in-depth cell response analysis.[20] In contrast to the strategies mentioned above, our system aims to provide 3D soft tissue mimic for true cell encapsulation.

Recently, Silva et al. (2018) published their development of metalloproteinase-1 degradable GG hydrogels, which were equipped with divinyl-sulfone groups to enable modular attachment of cell-adhesive peptides, namely, T1 and C16, both derived from ECM. According to the authors, elongation of the cells is achieved when coupled with a peptide sequence from the Cyr61/CCN1 protein at a concentration of 800 μM in the gel.[42]

### Structure analysis

In our project, the concentration of avidin coupled to gellan gum has been determined through fluorescence titration with a biotinylated dye. From the fluorescence titration we can conclude a concentration of 0.075 μM (L)/0.375 μM (H) avidin in 1 mg/mL NaGG solution.

In dry formulation (before dissolving and adding biotinylated species) 0.004 (L)/ 0.021 (H) mg/mg avidin:NaGG ratio. The functionalization yield is 21% (L) and 54% (H), respectively, as calculated from the total amount of CNCA used in the functionalization reaction.

Ferris et al. (2015) directly coupled RGD peptide to GG via carbodiimide coupling, report a functionalization yield of roughly 20% of carboxyl group concentration.[23] This peptide sequence (0.65 kDa) is significantly smaller than the protein avidin (57 kDa) and steric effects preventing higher coupling efficiency with carbodiimide strategy are plausible for avidin. As reported by Ferris et al. (2015), the purification of GG and the removal of divalent counterions increases activation yield with EDC and NHS, and thus convinced us to employ the same strategy.[23] The functionalization yield is further crucial for the number and density of available cell cues in the hydrogel matrix.

It has been shown that cells are sensitive to the presentation, spacing, and clustering of ligands in 2D cell culture systems and the interplay of substrate stiffness and availability of adhesion factors is well understood. In 2D, an RGD ligand spacing of less than 60 nm exhibited significantly improved adhesion and was found to be optimal.^4^ However, the reality for 3D matrices is less explored and likely more complex.

One of the core issues for this study was the nature of the binding of avidin to the polymer chain because a covalent attachment of avidin is substantial for the modular approach.^4,5,10^ In the literature, it has been shown that unattached compounds can also enhance cell response.^25^ However, anchoring the attachment cue to the network is required to generate the mechanotransduction effect, and the ECM receptors of the cell “pull” on the cue rather than the network. If the avidin were merely entrapped via unspecific binding and electrostatic interactions, the biotinylated cell cues would not appropriately convey the matrix stiffness to the cells. Further, the diffusion of the attachment cue out of the matrix system would be anticipated. Our analysis of the functionalized material with electrophoresis showed sufficiently that avidin is covalently tethered to the polymer network.

### Crosslinking and physical properties

Mechanical testing revealed that functionalization does not significantly alter the ability of the material to form gels. Forming true gels of NaGG-avd with the relevant mechanical properties for tissue engineering has proven to be uncomplicated and similar to other GG-based hydrogels.[25,43–45] The stiffness and mechanical behavior of the bulk hydrogel should mimic natural tissue, as it has been shown numerous times in the literature to influence, e.g., the differentiation of anchorage-dependent stem cells.[3,8] To prove their adequate mechanical properties, the loss modulus G” and storage modulus G’ determined through rheological assessment is often reported for hydrogels. The drawbacks of rheology include problematic sample preparation, complicated data interpretation, and GG is possibly too brittle a material to be analyzed through rheology.[24] Thus, in this study we employed compression testing of the hydrogel, and thereby benefitted from easy sample preparation and straight-forward data interpretation of the fracture point. The determination of the compression modulus is, however, challenging and prone to errors due to the softness of the material and instrument limitations as well as the unclear elastic region or the nonlinear elasticity of the hydrogel material. [46,47] Regardless of the above-mentioned limitations, compression testing offers an easy and fast method of comparison between different hydrogels and hydrogel compositions, especially when comparing different compositions using the exact same test parameters, or within the same study.

Because the goal of this study is to develop a non-specialized 3D hydrogel platform for cell culture, our system needs to have the ability to adapt not only to a wide range of biochemical cues, e.g. growth factors and peptides, but also a wide range of biophysical cues and mechanical stiffness requirements. Gellan gum based hydrogels have already been shown to provide a flexible platform and an adequate stiffness range which can be adjusted by polymer concentration and crosslinker type and concentration. Koivisto et al. (2017) demonstrate a linear correlation between crosslinker concentration and final stiffness of gellan gum hydrogels using the bioamines spermine and spermidine.[24]

It must be noted that the functionalization reaction uses carboxyl groups of glucuronic acid, which are also needed to form crystalline junction zones as crosslinking sites and to form the hydrogel. However, NaGG-avd forms true gels with very similar compression behavior to non-functionalized NaGG. This clearly indicates that a sufficient number of carboxyl groups is still available for crosslinking even after the avidin-functionalization. The addition of DMEM to the gels increases their fracture strength, which can be explained by the formation of tighter crystalline junction zones in the presence of higher cation concentration. Ions present in the swelling medium increase the crosslink density, cause densification, and thus increase the fracture strength of the hydrogels.

On a more practical note, the formed NaGG-avd hydrogels can be manipulated and moved with ease. The gelation time of the hydrogels has been determined to be between 30 seconds and one minute. This indicates a possibility to use the hydrogel as an injectable scaffold, which has not yet been further explored.

### Cell response

The functionalized material was tested as cell culture support, while using biotinylated RGD and biotinylated fibronectin for bioactive modification. All the required components are non-toxic and biocompatible. The crosslinking method is gentle and does not disturb cells because there are no side products of the crosslinking reaction. Further, the applied gel preparation achieves a true 3D structure, where the cells are homogeneously distributed throughout the hydrogel. This homogenous cell distribution is achieved by mixing the hydrogel sol with the cell suspension using a magnetic stirrer at 300 rpm, before casting the mixture to the wells of the well plate. Supported by the high viability results, this treatment does not harm the cells. Indeed, the short-term test with human WI-38 fibroblasts demonstrated high cell viability throughout different compositions.

GG has previously been shown to be suitable for soft tissue culture, but it is rather bioinert.[23–26,48] Here, we have shown that after functionalization, GG retains its good mechanical properties, such as soft tissue mimicking, while adding biofunctionalization.

A direct comparison of the cell culture results is challenging due to the ubiquity of cell lines and analysis methods used in the literature. Regarding the fibroblasts, we can point out similar viability of the same WI-38 cells studied by Pacelli et al. (2016) who report 74% viability with GG-based hydrogel after 4 days.[48] However, their cells were seeded in a 2D layer for 24 h more than in our study, and viability was asserted through optical density with a so-called neutral red assay. For 3D cultures, however, dye-based assays require optimization because the hydrogel affects both light propagation and the diffusion of the dye during incubation. In the GG-based hydrogel study by da Silva et al. (2018) mentioned earlier[42], image analysis was used to determine the cell viability of human umbilical vein endothelial cells (HUVECs) in thiol-functionalized gellan gum. The authors found a 65% viability of HUVECs after 3 days encapsulated in the hydrogel. Overall, our observation of WI-38 fibroblast viability was between 85% and 90% in different formulations of NaGG-avd, which exceeds or at least matches the values found in the literature for GG-based hydrogels. Therefore, the excellent biocompatibility of NaGG-avd can be affirmed.

There appears to be no overall visible difference in WI-38 cell viability between 2D and 3D conditions, with live and dead cells being present in either culture method with substantial standard deviation. 2D culture promotes the growth of cell aggregates and the cells possibly migrate to form larger clusters, possibly due to the inert properties of the hydrogel surface. When encapsulated in 3D, the cells seemingly cannot migrate, and are therefore limited to grow within the hydrogel in the area of initial original deposition. There is no visible difference in 3D cell distribution between the different biotinylated compounds added to NaGG-avd. Overall, no statistical significance in cell spreading of could be found between all tested gellan-gum compositions (GG and NaGG-avd samples). From Fig 6B a trend is visible favoring the avidin-functionalized samples, however the large standard deviation prevents any decisive statement. A higher degree of cell adhesion and spreading would have been anticipated from the presence of ECM derived factors. However, this effect could be explained by the presence of serum proteins in the culture medium that compete with the cells to reach the provided attachment sites. This is largely due to the Vroman effect[49] that describes the mobility-dependent adsorption of proteins onto hydrophilic surfaces. Thus, serum components, such as albumin, fibrinogen, and fibronectin, attach faster to the biomaterial than the cells and favoring them may prevent the cell receptors from recognizing any available cell adhesive cues covalently bound to the biomaterial. In order to avoid the Vroman effect, some studies have successfully used serum starving conditions to show enhanced cell attachment and survival in biomaterials that contain cell adhesive cues.[50,51]

The same tendency of cell behavior was visible in hBMSC 3D culture in our functionalized gellan gum. NaGG-avd had been equipped with biotinylated RGD to stimulate cell adhesion because RGD is known to be a crucial and prevalent peptide sequence for cell integrin recognition of ECM. RGD has been used frequently for BMSC studies and positive effects have been reported in the literature[11,12,52], and thus was chosen here as a model cell adhesive site. We can correlate our findings to a study by Anjum et al. (2016) who cultured hBMSC over 14 days in their chondroitin sulfate-poly(ethylene glycol) (PEG) hydrogel and found 80% to 95% viability between different compositions.[53] The LIVE/DEAD^®^ stained images appear similar in viability to our findings and interestingly also show a similar obfuscation with time. Similarly, Blache et al. (2016) used RGD-functionalized PEG-based hydrogel and co-cultured HUVEC+hMSC. The authors reported good cell viability and definite cell elongation with a three-times higher cell concentration compared with our study.[11] Another comparable study has been published by Tsaryk et al. (2014) using ionically crosslinked methacrylated GG (iGG-MA) with human mesenchymal stem cells (hMSC) and nasal chondrocytes. [54] They achieved good viability and adequate differentiation towards chondrogenic and osteogenic lineages. No attachment factors had been added for this encapsulation study, and the differentiation is steered solely through medium composition. After 14 days encapsulated in iGG-MA, the hMSC showed a round shape, while the hydrogel opacity increased, and the confocal microscope image clarity deteriorated similar to our study. In summary, different approaches to design hydrogels for the encapsulation of human mesenchymal stem cells have been presented in the literature.

Our proof-of-concept with avidin-functionalized NaGG provides an easy modification of hydrogel material for cell culture applications. In the laboratory, the hydrogel is rather simple to handle and requires the combination of only three components. The persisting problem in the presented results is the small amount of avidin coupled to the gellan gum network. This in turn leads to a rather low number of bioactive cues available for the cells. As a brief comparison, Broguiere et al. (2018) report that a fibrin hydrogel at 3 mg/mL provides a sufficient concentration of RGD binding sites at 75 μM, whereas our results show a concentration of 0.3 μM bRGD in the final hydrogel (Table 1).[55] Further research is therefore needed to increase functionalization degree and overcome the problem of the low number of bioactive cues in the hydrogel. A range of other functionalization reaction is conceivable, such as bio-orthogonal click reactions.[56] However, an interference of high functionalization degree with crosslinking capability must be considered. Another prospect could be the functionalization of the polymer chain end-groups to avoid blocking the carboxyl groups of gellan gum, as proposed by Bondalapati et al. (2014).[57] While considering an alternative functionalization reaction, the carbodiimide strategy will always have the advantage of being well-known and heavily used in the literature[14], and thus we can rely on its efficacy. As an example, the end-group modification strategy would have to be tested and refined for gellan gum and avidin, and additionally the cytotoxic substance aniline is used as a catalyst, which may prove problematic.

**Table 1:** Component concentration of hydrogel composition.

Component	Mass of component	Weight percent	Concentration
**Avidin (L)/(H)**	0.02/ 0.11 mg	0.002/0.011 wt%	0.375/ 1.875 μM
**NaGG**	5 mg	0.50%	10 μM
**bRGD/bFn**	0.3 x10^-3^/0.03 mg	0.03x10^-3^/0.003 wt%	0.27/ 0.08 μM
**H_2_O**	995 mg	99.5%	

With the proposed avidin-biotin modification system, the modularity hinges solely on the availability of the biotinylated compounds. There is a multitude of these compounds already commercially available, but they can also be synthesized by biotinylating the desired compound, if such a compound is not yet available. As proof-of-concept, these results underline the viability of stromal cells in a gellan gum-based 3D hydrogel cell culture system. Our approach is suitable for disease modeling applications due to its capacity to image cells inside the hydrogel and the ability to control the 3D biochemical environment through avidin-biotin modularity. Future work will focus on the effect of biotinylated species and quantitative assessment of stromal cells encapsulated in our modular hydrogel.

## Conclusions

Gellan gum was successfully functionalized with the avidin protein (charge neutralized chimeric avidin). This functionalized hydrogel provides a general, modular platform capable of tethering biotinylated compounds and aiding the attachment and proliferation of cells in 3D. Contrasting the common strategy of direct attachment, our modular hydrogel is intended to be off-the-shelf ready for any cell culture application, while still being consistent in physical properties. The current proposed avidin functionalization system is not targeted at a specific cell type or application, but rather intended to form the base material for further studies. Further modification by the addition of a cell type specific biotinylated compound is necessary to enhance the positive cell response. The presented data using two different cell types support that the avidin-modified gellan gum is a useful tool applicable for cell culture. The hydrogel is biocompatible, sterilizable, and retains adequate mechanical properties and stability over several weeks. This long-term stability is essential for disease or tissue models involving cell types with a slow metabolism and development, such as bone and cartilage derived cells.

## Supporting information

S1 Appendix ICP-OES data. Elemental composition of counterions in commercial Gelzan^TM^ (GG) and purified product (NaGG) has been determined with ICP-OES. The ion concentration of the purified product matches values found in literature (24,28).
(PDF)

S2 Appendix SDS PAGE. Full uncropped image of sodium dodecyl sulfate polyacrylamide gel electrophoresis (SDS PAGE) blot.
(PDF)

S3 Appendix Compression data of functionalized and non-functionalized samples. Data are the same as presented in Fig 4B and are presented as means ± SD. The graphs (stress vs. strain) show all compression curves, from which the fracture points are averaged. The photograph shows cylindrical hydrogel samples, cell-free but incubated in DMEM.
(PDF)

S4 Appendix Swelling degree of NaGG-avd(L) hydrogel samples. Hydrogels were prepared in Eppendorf tubes with either SPD or CaCl_2_ as crosslinker and the initial mass of each sample was taken. Swelling media, either PBS or DMEM, were added on top after the gels had set and the samples were incubated at 37°C up to 3 weeks. Swelling degree was calculated through monitoring change in mass according to Eq (3). Data are shown as means ± SD (n = 3).
(PDF)

S5 Appendix Cell viability from particle counting. Averaged values of particle counting algorithm results for all LIVE/DEAD^®^ images. The bar graph shows average values of image-by-image comparison for ‘total area’ value.
(PDF)

S6 Appendix Flow cytometry surface markers analysis results of hBMSCs. n = 1. The flow cytometry analysis confirmed the mesenchymal origin of the hBMSCs.
(PDF)

S7 Appendix Cytoskeleton images of hBMSC after 21 days. Blue channel = cell nuclei (DAPI) and red channel = actin filaments (TRITC-phalloidin). (A) Cells in NaGG and (B) NaGG-avd+bRGD.
(PDF)

S8 Appendix Washing test and immunofluorescence straining. Description of washing test for hydrogel samples formed with NaGG and NaGG-avd using biotinylated fibronection (bFn). Immuncytochemistry staining for fibronectin shows 4-fold retention of fibronectin in avidin-functionalized gel.
(PDF)

S9 Appendix Microscope images of WI-38 cell culture experiment for counting analysis. Exemplary images of 3D samples between day 0 (light microscope) and day 3 (LIVE/DEAD^®^ fluorescence images) for comparison.
(PDF)
