# Supplementary material for: Design of modular gellan gum hydrogel functionalized with avidin and biotinylated adhesive ligands for cell culture applications
Source: PLoS One. 2019 Aug 30;14(8):e0221931. doi: 10.1371/journal.pone.0221931 (PMC6716642; doi:10.1371/journal.pone.0221931)
Supplement: S1 Appendix — Elemental composition of counterions in commercial GelzanTM (GG) and purified product (NaGG) has been determined with ICP-OES. The ion concentration of the purified product matches values found in literature (24,28). (PDF) [file pone.0221931.s001.pdf]

Christine Gering, Janne T. Koivisto, Jenny Parraga, Jenni Leppiniemi, Kaisa Vuornos, Vesa P. Hytönen, Susanna Miettinen, and Minna Kellomäki

S1 Appendix. ICP-OES data.

Elemental composition of counterions in commercial Gelzan^{TM} (GG) and purified product (NaGG) has been determined with ICP-OES. The ion concentration of the purified product matches values found in literature (24,28).

	**GG**	**NaGG**	**Reference NaGG (24,28)**
Ca [wt%]	0.29%	0.08%	0.04 – 0.06%
K [wt%]	4.86%	0.25%	1.00 - 1.02%
Mg [wt%]	0.10%	0.02%	0.03%
Na [wt%]	0.49%	2.73%	2.50- 2.51%

References

24. Ferris CJ, Stevens LR, Gilmore KJ, Mume E, Greguric I, Kirchmajer DM. Peptide modification of purified gellan gum. Journal of Materials Chemistry B 2015;3(6):1106-1115.

28. Doner LW. Rapid purification of commercial gellan gum to highly soluble and gellable monovalent cation salts. Carbohydrate Polymers 1997;32(3):245-247.
