# Supplementary material for: Design of modular gellan gum hydrogel functionalized with avidin and biotinylated adhesive ligands for cell culture applications
Source: PLoS One. 2019 Aug 30;14(8):e0221931. doi: 10.1371/journal.pone.0221931 (PMC6716642; doi:10.1371/journal.pone.0221931)

Christine Gering, Janne T. Koivisto, Jenny Parraga, Jenni Leppiniemi, Kaisa Vuornos, Vesa P. Hytönen, Susanna Miettinen, and Minna Kellomäki

S2 Appendix. SDS-PAGE (Sodium dodecyl sulfate polyacrylamide gel electrophoresis)

Full uncropped image of urea sodium dodecyl sulfate polyacrylamide gel electrophoresis (SDS PAGE) blot. Wells 1,3,5 and 7 are loaded with ruler (PageRuler^{TM}, unstained protein ladder, ThermoScientific), NaGG-avd, NaGG and avidin mixture, and pure avidin (CNCA) respectively. All avidin-containing samples were stabilized with the addition of biotin to a final concentration of 85 µg/mL biotin.. Every second well was left empty to achieve good spacing between samples to prevent mixing of bands. The red areas in the gel figure represent areas with overexposure.

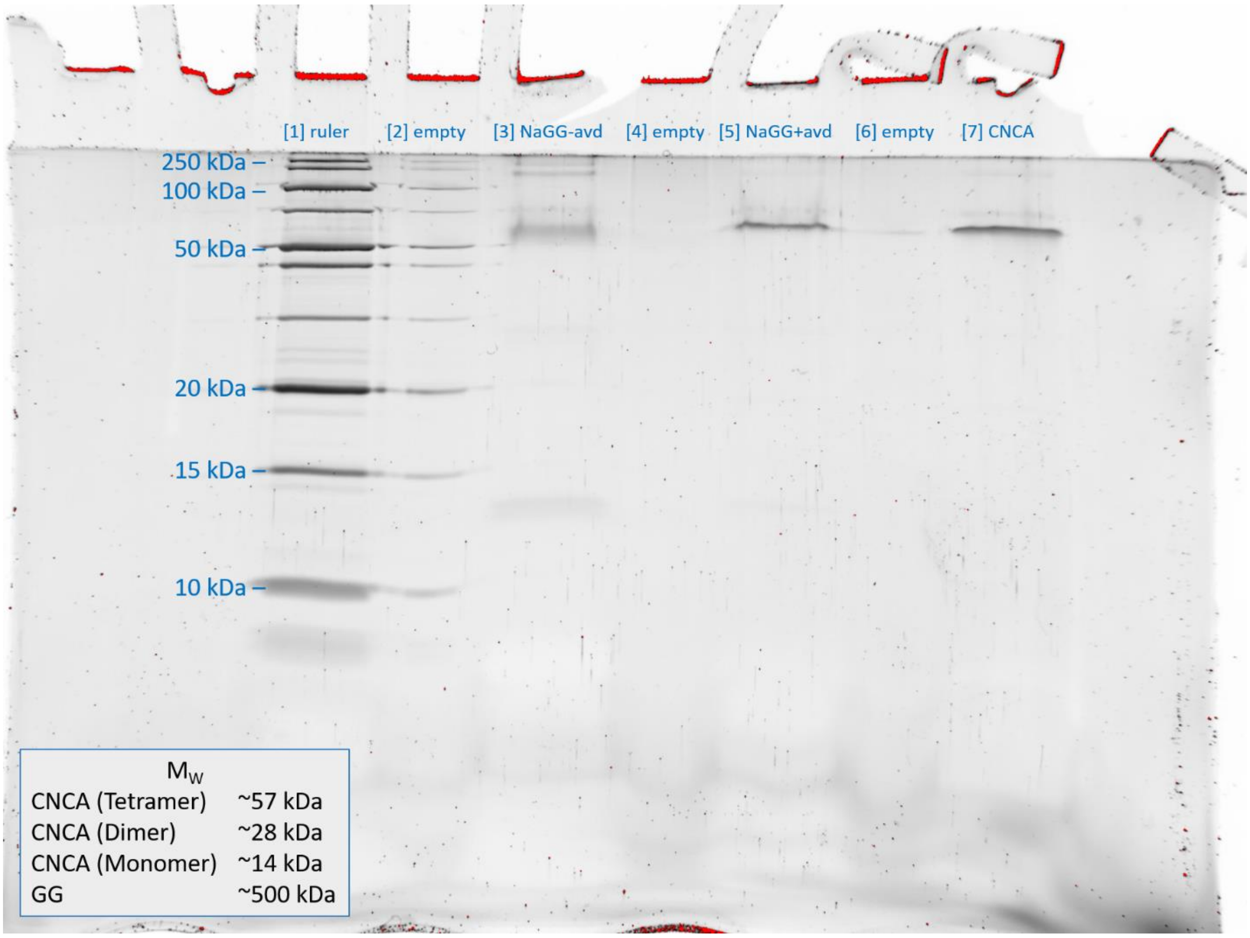

Supplement: S2 Appendix — Full uncropped image of sodium dodecyl sulfate polyacrylamide gel electrophoresis (SDS PAGE) blot. (PDF) [file pone.0221931.s002.pdf]
