# Supplementary material for: Design of modular gellan gum hydrogel functionalized with avidin and biotinylated adhesive ligands for cell culture applications
Source: PLoS One. 2019 Aug 30;14(8):e0221931. doi: 10.1371/journal.pone.0221931 (PMC6716642; doi:10.1371/journal.pone.0221931)

Christine Gering, Janne T. Koivisto, Jenny Parraga, Jenni Leppiniemi, Kaisa Vuornos, Vesa P. Hytönen, Susanna Miettinen, and Minna Kellomäki

S3 Appendix. Compression data and curves of functionalized and non-functionalized samples.

Data are the same as presented in Fig. 4B and are presented as means ± SD. The graphs (stress vs. strain) show all compression curves, from which the fracture points are averaged. The photograph shows cylindrical hydrogel samples, cell-free but incubated in DMEM.

Sample description	Fracture Strength [kPa]	Fracture Strain [%]	*n*
NaGG + SPD	4.3 ± 1.1	32.7 ± 6.5	5
NaGG + $CaCl_2$	5.1 ± 0.8	45.5 ± 1.0	5
NaGG-avd (L) + SPD	5.5 ± 0.7	35.9 ± 4.0	5
NaGG-avd (L) + $CaCl_2$	8.6 ± 1.0	45.7 ± 2.2	5
NaGG-avd (H) + SPD	6.4 ± 1.2	36.5 ± 5.6	4
NaGG-avd DMEM on gel	11.5 ± 0.8	37.6 ± 3.5	5

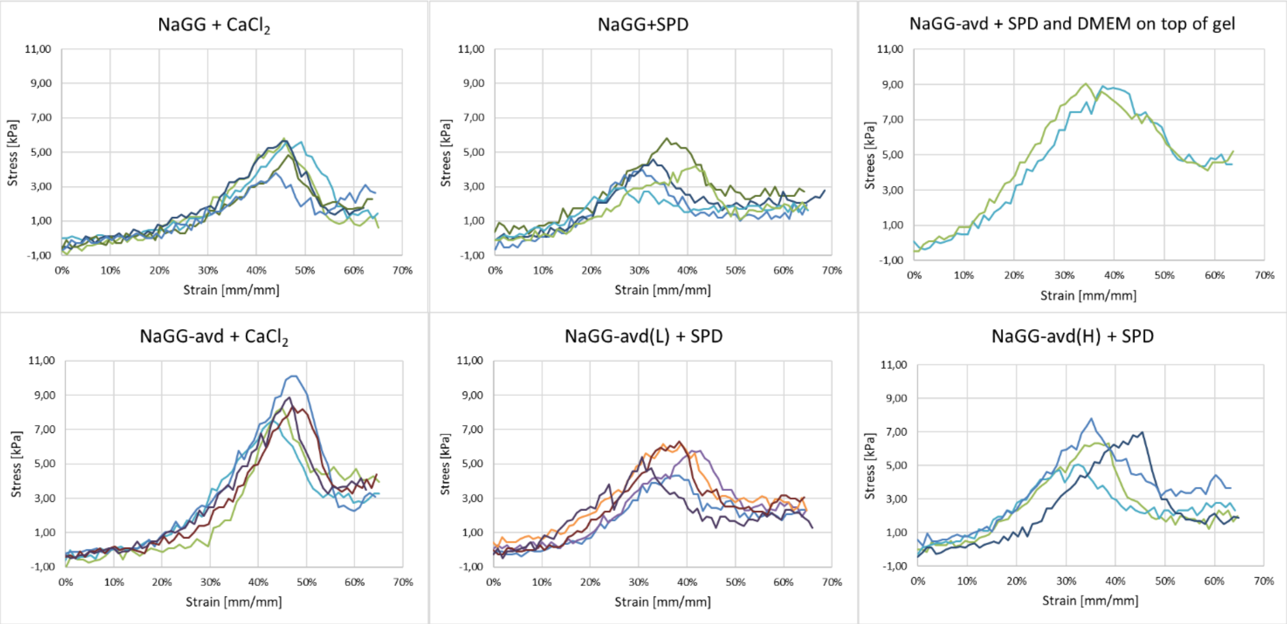

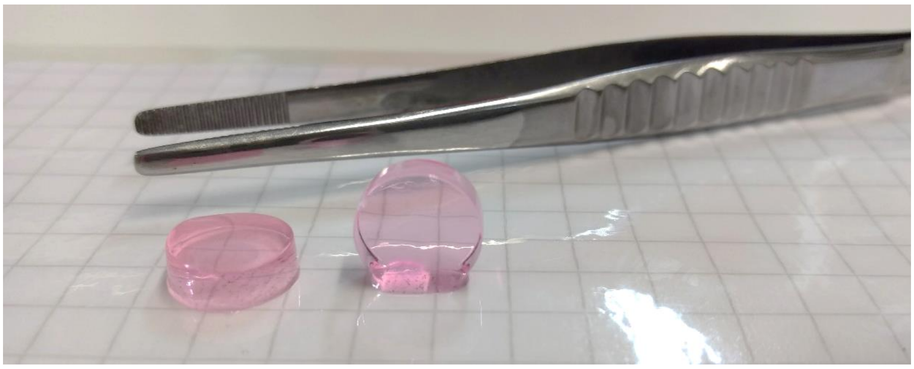

Supplement: S3 Appendix — Data are the same as presented in Fig 4B and are presented as means ± SD. The graphs (stress vs. strain) show all compression curves, from which the fracture points are averaged. The photograph shows cylindrical hydrogel samples, cell-free but incubated in DMEM. (PDF) [file pone.0221931.s003.pdf]
