# Supplementary material for: Design of modular gellan gum hydrogel functionalized with avidin and biotinylated adhesive ligands for cell culture applications
Source: PLoS One. 2019 Aug 30;14(8):e0221931. doi: 10.1371/journal.pone.0221931 (PMC6716642; doi:10.1371/journal.pone.0221931)

S4 Appendix. Swelling degree of NaGG-avd(L) hydrogel samples.

Hydrogels were prepared in Eppendorf tubes with either SPD or $CaCl_2$ as crosslinker and the initial mass of each sample was taken. Swelling media, either PBS or DMEM, were added on top after the gels had set and the samples were incubated at 37ºC up to 3 weeks. Swelling degree was calculated through monitoring change in mass according to **equation (3)**. Data are shown as means ± SD (n = 3).

$$swelling\ degree = \frac{swollen\ weight - dry\ weight}{dry\ weight} \times 100\% \qquad (3)$$

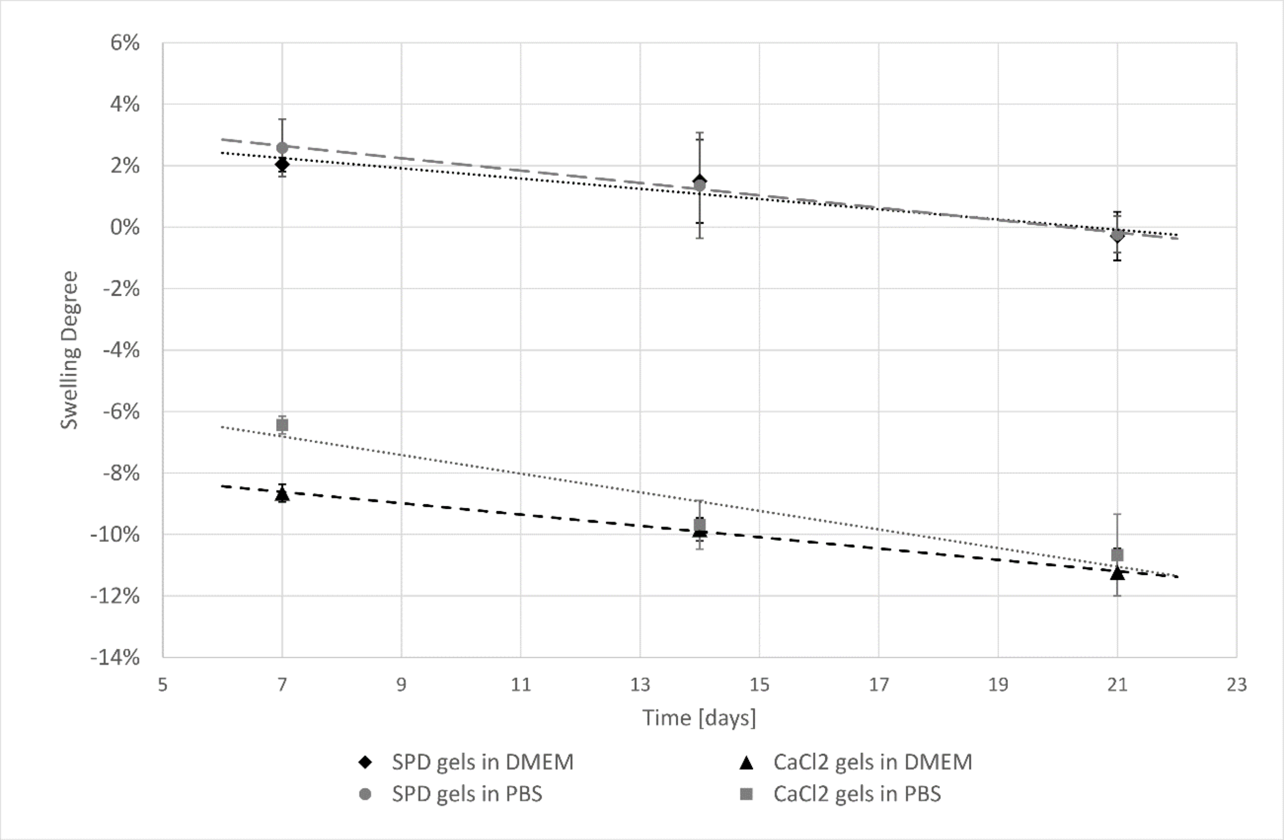

Supplement: S4 Appendix — Hydrogels were prepared in Eppendorf tubes with either SPD or CaCl2 as crosslinker and the initial mass of each sample was taken. Swelling media, either PBS or DMEM, were added on top after the gels had set and the samples were incubated at 37°C up to 3 weeks. Swelling degree was calculated through monitoring change in mass according to Eq (3). Data are shown as means ± SD (n = 3). (PDF) [file pone.0221931.s004.pdf]
