# Supplementary material for: Design of modular gellan gum hydrogel functionalized with avidin and biotinylated adhesive ligands for cell culture applications
Source: PLoS One. 2019 Aug 30;14(8):e0221931. doi: 10.1371/journal.pone.0221931 (PMC6716642; doi:10.1371/journal.pone.0221931)

Christine Gering, Janne T. Koivisto, Jenny Parraga, Jenni Leppiniemi, Kaisa Vuornos, Vesa P. Hytönen, Susanna Miettinen, and Minna Kellomäki

S5 Appendix. Cell viability from particle counting

Averaged values of particle counting algorithm results for all LIVE/DEAD® images. The bar graph shows average values of image-by-image comparison for 'total area' value.

		LIVE				DEAD			
		Particle count	Total area	Average size	% area	Particle count	Total area	Average size	% area
**2D**	Control (TCP)	498.4	34.351	0.070	51.148	28.8	0.050	0.002	0.075
	GG	29.4	1.073	0.038	1.597	53.8	0.171	0.003	0.255
	NaGG	10.9	0.624	0.066	0.930	23.9	0.099	0.004	0.148
	NaGG-avd + bFN	63.8	4.175	0.053	6.216	84.2	0.183	0.002	0.273
	NaGG-avd + biotin	75.7	1.130	0.015	1.683	51.6	0.169	0.004	0.251
	NaGG-avd + bRGD	22.3	1.376	0.066	2.049	44.5	0.099	0.002	0.147
**3D**	GG	29.3	0.679	0.024	1.012	20.8	0.066	0.003	0.099
	NaGG	125.9	1.721	0.014	2.563	15.8	0.085	0.005	0.127
	NaGG-avd + bFN	256.3	2.395	0.009	3.566	113.9	0.352	0.003	0.524
	NaGG-avd + biotin	208.8	1.898	0.010	2.826	36.7	0.291	0.010	0.433
	NaGG-avd + bRGD	253.9	2.668	0.011	3.972	35.1	0.164	0.005	0.244

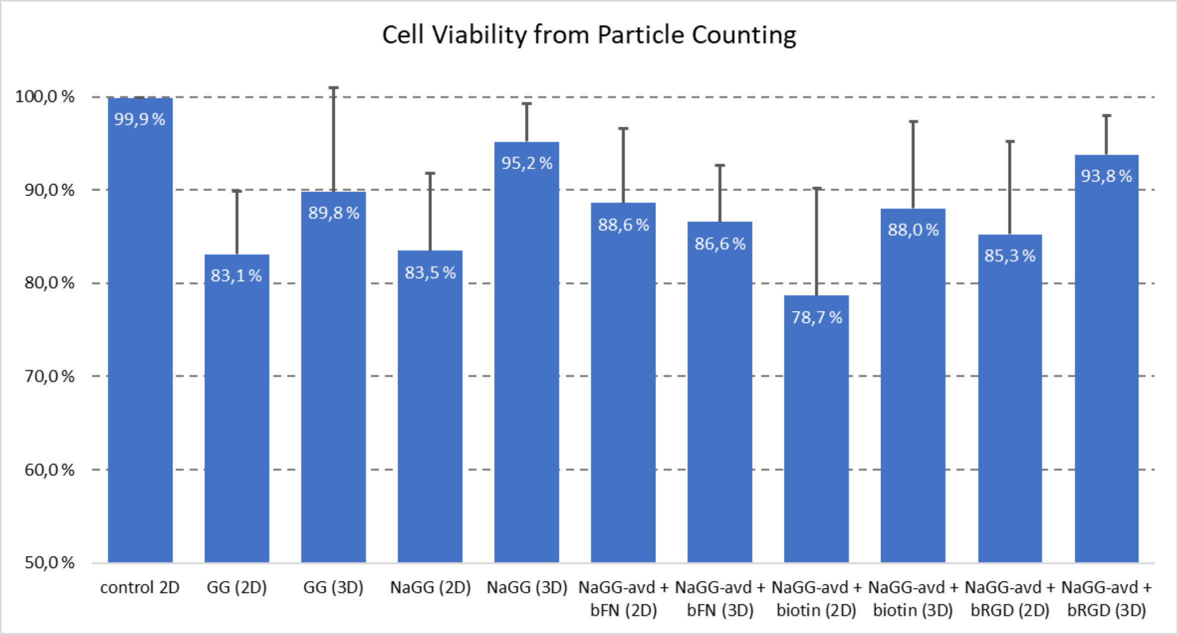

Supplement: S5 Appendix — Averaged values of particle counting algorithm results for all LIVE/DEAD® images. The bar graph shows average values of image-by-image comparison for ‘total area’ value. (PDF) [file pone.0221931.s005.pdf]
