# Supplementary material for: Design of modular gellan gum hydrogel functionalized with avidin and biotinylated adhesive ligands for cell culture applications
Source: PLoS One. 2019 Aug 30;14(8):e0221931. doi: 10.1371/journal.pone.0221931 (PMC6716642; doi:10.1371/journal.pone.0221931)
Supplement: S6 Appendix — n = 1. The flow cytometry analysis confirmed the mesenchymal origin of the hBMSCs. (PDF) [file pone.0221931.s006.pdf]

S6 Appendix. Flow cytometry surface markers analysis results of hBMSCs

Following stem cell isolation, the undifferentiated human bone marrow stem cells (hBMSCs) of passage 3 were characterized. Briefly, hBMSCs were analyzed for the cell surface markers with flow cytometry (FACSAria; BD Biosciences, Franklin Lakes, NJ, USA). The following fluorophore-conjugated monoclonal antibodies were used: anti-CD3-PE, anti-CD14-PECy7, anti-CD19-PE-Cy7, anti-CD45RO-APC, anti-CD73-PE, anti-CD90-APC (BD Biosciences), anti-CD11a-APC, anti-CD105-PE (R&D Systems, Minneapolis, MN, USA), CD34-APC, and anti-HLA-DR-PE (ImmunoTools, Friesoythe, Germany). Flow cytometry analysis was performed with 10,000 cells per sample, and the positive expression was defined as a level of fluorescence 99 % greater than that of the corresponding unstained cell sample (56, 57). In the analysis, the majority of hBMSCs expressed the surface markers CD73, CD90, CD105, and HLA-DR while expression of CD3, CD11a, CD14, CD19, CD34, and CD45 was low (Table S3).

Flow cytometry surface markers analysis results of hBMSCs. n=1. The flow cytometry analysis confirmed the mesenchymal origin of the hBMSCs.

Antigen	Surface protein	
CD 3	T cell signal transduction	1.0
CD 11a	Cell interactions and T cell mediated killing	0.4
CD 14	Innate immune response to bacterial lipopolysaccharide	4.6
CD 19	B lymphocyte-lineage differentiation antigen	3.4
CD 34	Sialomucin-like adhesion molecule	2.3
CD 45	Leukocyte common antigen	6.4
CD 73	Ecto-5'-nucleotidase	89.1
CD 90	Thy-1 (T cell surface glycoprotein)	81.5
CD 105	SH-2, endoglin	86.5
HLA-DR	Major histocompatibility class II antigens	76.4

References

55. M.J. Jaroszeski & G. Radcliff, Fundamentals of flow cytometry, Mol.Biotechnol., 11 No. 1 (1999) 37-53. 10.1007/BF02789175.

56. J. Picot, C.L. Guerin, C. Le Van Kim, C.M. Boulanger, Flow cytometry: retrospective, fundamentals and recent instrumentation, Cytotechnology, 64 No. 2 (2012) 109-130. 10.1007/s10616-011-9415-0; 10.1007/s10616-011-9415-0.
