# Supplementary material for: Design of modular gellan gum hydrogel functionalized with avidin and biotinylated adhesive ligands for cell culture applications
Source: PLoS One. 2019 Aug 30;14(8):e0221931. doi: 10.1371/journal.pone.0221931 (PMC6716642; doi:10.1371/journal.pone.0221931)

Christine Gering, Janne T. Koivisto, Jenny Parraga, Jenni Leppiniemi, Kaisa Vuornos, Vesa P. Hytönen, Susanna Miettinen, and Minna Kellomäki

S7 Appendix. Cytoskeleton images of hBMSC after 21 days.

Blue channel = cell nuclei (DAPI) and red channel = actin filaments (TRITC-phalloidin).

(A) Cells in NaGG and (B) NaGG-avd+bRGD.
(C) Background images of cell-free hydrogels stained with DAPI and phalloidin

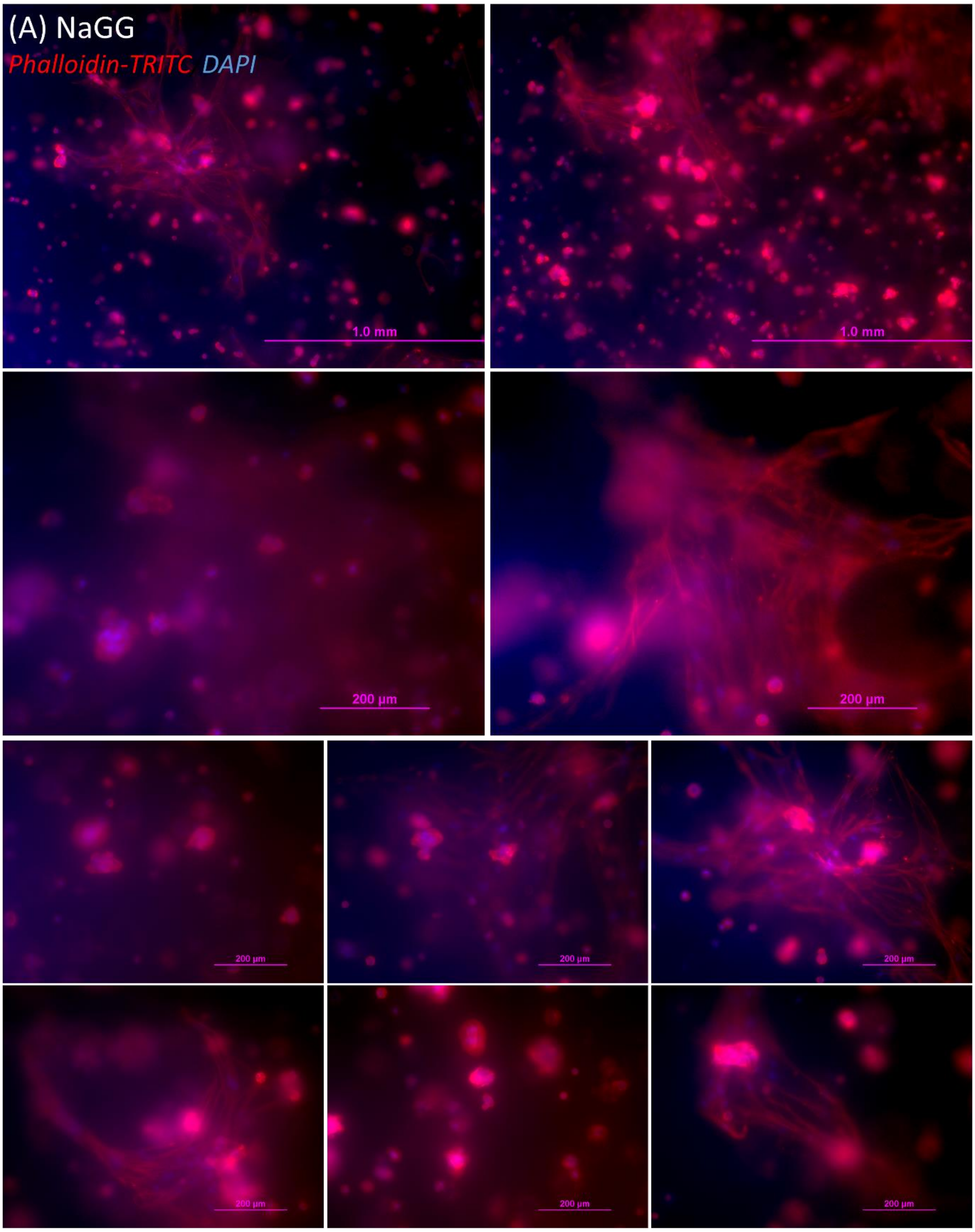

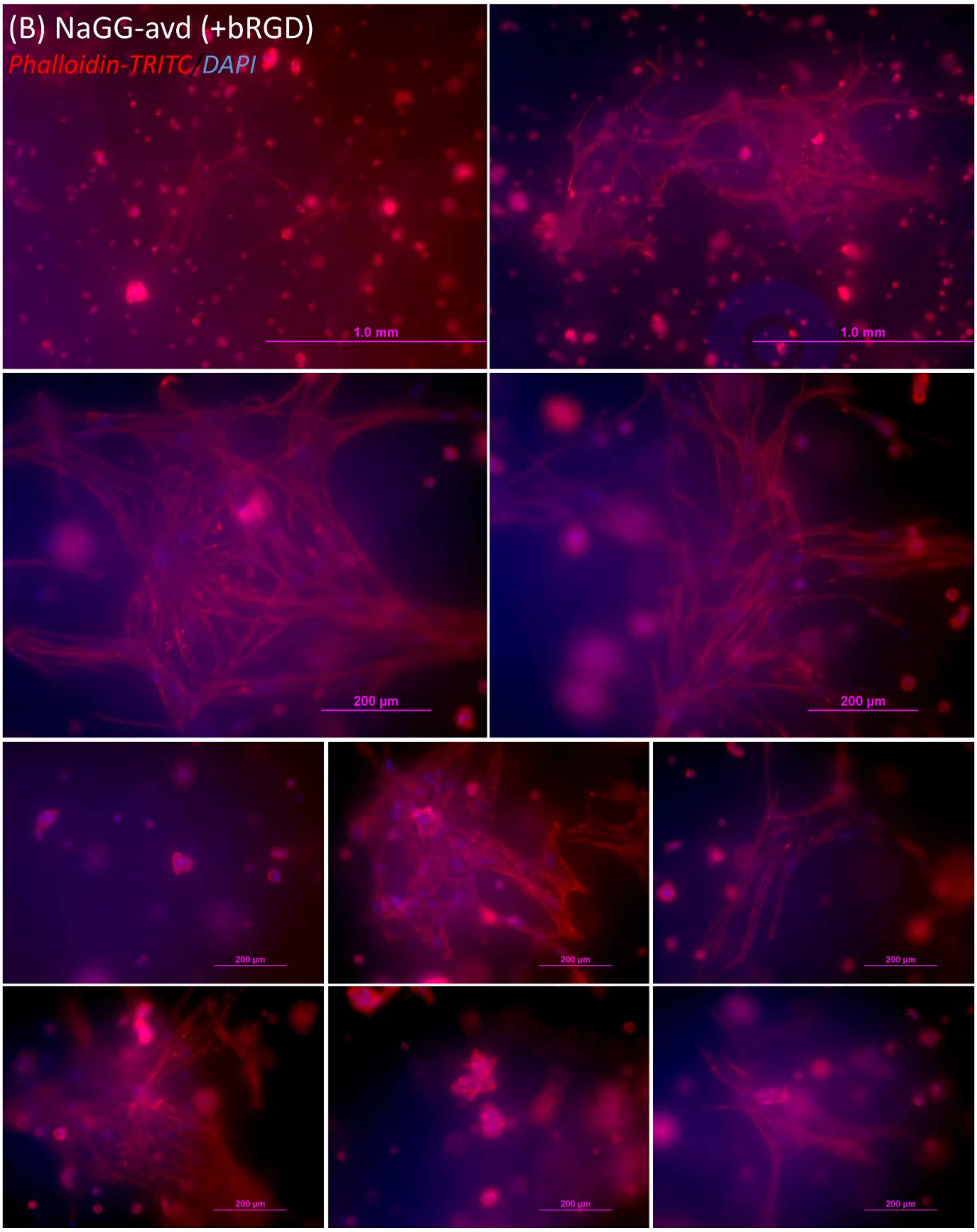
(B) NaGG-avd (+bRGD)
Phalloidin-TRITC/DAPI
1.0 mm
1.0 mm
200 µm
200 µm
200 µm
200 µm
200 µm
200 µm
200 µm
200 µm

(C) Background images of cell-free hydrogels stained with DAPI and phalloidin

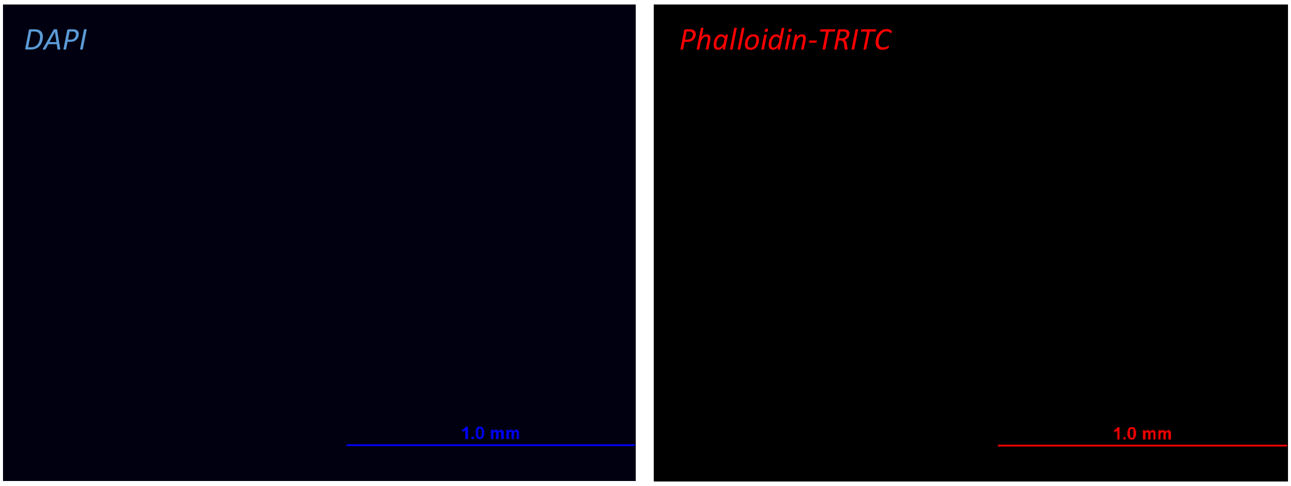

Supplement: S7 Appendix — Blue channel = cell nuclei (DAPI) and red channel = actin filaments (TRITC-phalloidin). (A) Cells in NaGG and (B) NaGG-avd+bRGD. (PDF) [file pone.0221931.s007.pdf]
