# Supplementary material for: Design of modular gellan gum hydrogel functionalized with avidin and biotinylated adhesive ligands for cell culture applications
Source: PLoS One. 2019 Aug 30;14(8):e0221931. doi: 10.1371/journal.pone.0221931 (PMC6716642; doi:10.1371/journal.pone.0221931)

S8 Appendix. Washing test and immunofluorescence straining

Hydrogels were prepared from NaGG (5 mg/mL) and NaGG-avd(H) (5 mg/mL) with bFN (2.52 mg/mL) and SPD (0.5 mg/mL) in a 5:0.065:1 ratio and incubated overnight. The "wash" samples were treated with PBS (10 min) and 0.1% triton X100 in PBS (3x 15 min) consequently. To stain for fibronectin, all samples were then blocked (1 h in 10% NDS, 0.1% triton X100, 1%BSA in PBS), washed (10 min 10% BSA, 1%NDS in PBS), treated with primary antibody (over 5 days at +4°C, rabbit anti-fibronectin, 1:250 in washing solution), washed again (1% BSA in PBS), and finally stained with secondary antibody (overnight at +4°C, Alexa 488 donkey anti-rabbit, 1:800 in washing solution).

The hydrogels were removed from the mold and placed on a glass cover slip to analyze with confocal microscope by using z-stack analysis that can penetrate 30µm into the gel surface. The microscope settings (pinhole, laser power, etc.) were kept constant throughout all measurements. For evaluation, the mean intensity of the stack was compared and is shown in the graph below. Data and error bars represent three measurements at different areas in one sample.

b-Fn with A488 immunostain

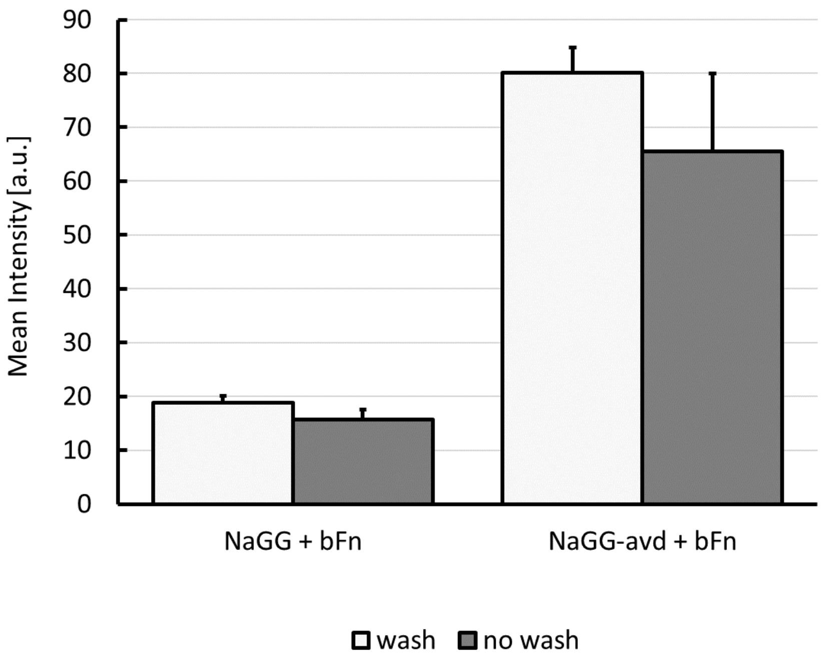

Supplement: S8 Appendix — Description of washing test for hydrogel samples formed with NaGG and NaGG-avd using biotinylated fibronection (bFn). Immuncytochemistry staining for fibronectin shows 4-fold retention of fibronectin in avidin-functionalized gel. (PDF) [file pone.0221931.s008.pdf]
