# Supplementary material for: Design of modular gellan gum hydrogel functionalized with avidin and biotinylated adhesive ligands for cell culture applications
Source: PLoS One. 2019 Aug 30;14(8):e0221931. doi: 10.1371/journal.pone.0221931 (PMC6716642; doi:10.1371/journal.pone.0221931)

S9 Appendix. Microscope images of WI-38 cell culture experiment for counting analysis

Cell culture experiment details: cell density: 2D = 63 000 cells/cm^2, 3D = 950 000 cells/mL. NaGG-avd compostion: (1) biotin (0.17 mg/mL in 50 mM sodium-phosphate, 100 mM NaCl buffer, pH 7), (2) biotinylated cyclic RGD (cyclo[Arg-Gly-Asp-D-Phe-Lys(Biotin-PEG-PEG)] 0.1 mg/mL in H_2O, 0.3 µg/mL in final gel), (3) biotinylated human fibronectin (bFN, 2.52 mg/mL, 33 µg/mL in final gel. Cultured in WI-38 medium over 3 days.

Left: light microscope images after encapsulation. Objective 4x
Right: Live/Dead ® stained images after 3 days. Objective 4x

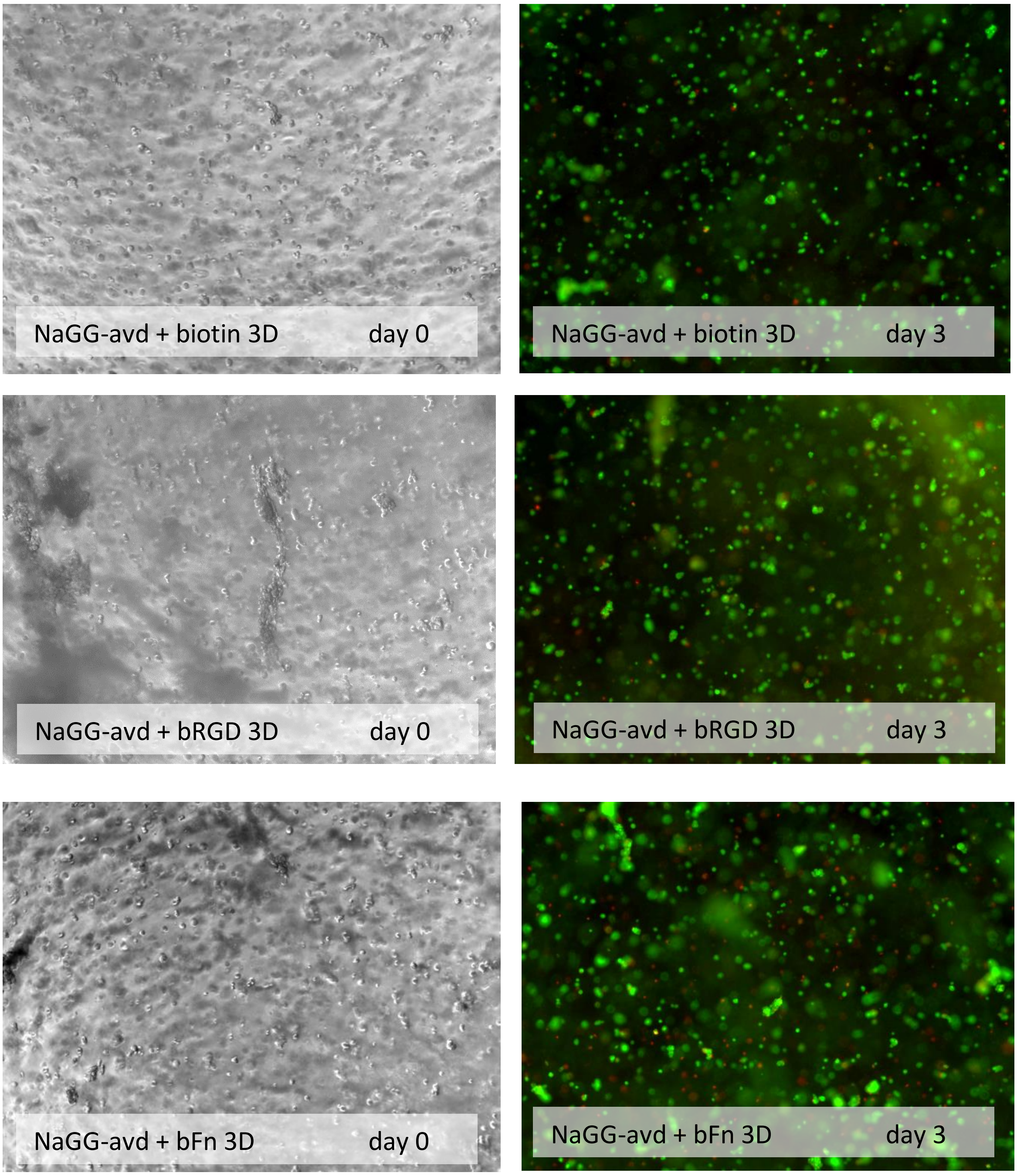

Supplement: S9 Appendix — Exemplary images of 3D samples between day 0 (light microscope) and day 3 (LIVE/DEAD® fluorescence images) for comparison. (PDF) [file pone.0221931.s009.pdf]
